# Proteins from Avastin® (bevacizumab) Show Tyrosine Nitrations for which the Consequences Are Completely Unclear

**DOI:** 10.1371/journal.pone.0034511

**Published:** 2012-04-16

**Authors:** Jia Wan, Edina Csaszar, Wei-Qiang Chen, Kongzhao Li, Gert Lubec

**Affiliations:** 1 Department of Pediatrics, Medical University of Vienna, Vienna, Austria; 2 Mass Spectrometry Facility, Max F. Perutz Laboratories, Vienna, Austria; Medical University of Vienna, United States of America

## Abstract

Avastin® (bevacizumab) is a protein drug widely used for cancer treatment although its further use is questionable due to serious side effects reported. As no systematic proteomic study on posttranslational modifications (PTMs) was reported so far, it was the aim of the current study to use a gel-based proteomics method for determination of Avastin®-protein(s).

Avastin® was run on two-dimensional gel electrophoresis (2-DE), spots were picked, followed by multi-enzyme in-gel digestion. Subsequently, the resulting peptides and posttranslational modifications were identified by mass spectrometry (nano-LC-ESI-MS/MS; HCT and LTQ Orbitrap MS). Heavy and light chains were observed and the 9 spots that were picked from 2DE-gels were identified as bevacizumab with high sequence coverage. MS/MS results showed multiple tyrosine nitrations on the Avastin® light and heavy chains that were either represented as nitrotyrosine or as aminotyrosine, which was shown to be generated from nitrotyrosine under reducing conditions. Protein nitration is known to significantly change protein functions and interactions and it may well be that some of the adverse effects of the protein drug Avastin® may be due to this PTM, which may have been generated during production- thus, nitration of Avastin® is a challenge for the pharmaceutical industry.

## Introduction

Avastin® (bevacizumab) is an antibody widely used in antitumor therapy with the underlying principle of neutralizing vascular endothelial growth factor (VEGF) thus moderating tumor growth. Although no final assessment of the effectiveness of bevacizumab in large cohorts of cancer patients has been published, it can be considered a therapeutic antibody with high potential [1]-[5].

Fatal adverse effects, however, have been reported in tumor patients treated with this protein drug and in according to a meta-analysis, bevacizumab therapy was associated with increased treatment-related mortality [6].

More specifically, bevacizumab treatment may significantly increase the risk of serious hemorrhage, hypertension, proteinuria, cardiac toxicity, vascular thromboembolism, gastrointestinal, dermatological and endocrine toxicity in cancer patients [7]-[15]. According to the statement by Stone et al. [10], toxicity management in treatment with anti-angiogenic agents has not been an endpoint in most studies carried out up to now and there is therefore a fundamental need for investigations that will generate more evidence-based practice guidelines.

Vascular side effects may be readily assigned to biological activity of VEGF inhibition directly whereas others may be due to other mechanisms, including probable modifications of this humanized antibody. No information on Avastin® protein modifications is available so far and the only systematic mass spectrometrical approach analysing Avastin® was not designed to characterise protein modifications but rather to determine stability [16].

The absence of this information formed the rationale for the current study with the aims to identify and characterise Avastin protein and protein modifications in order to form the basis for studies linking protein modifications to adverse side effects. And indeed, heavy tyrosine nitration, known to modify protein properties and functions, was observed in the commercially available medical product.

## Materials and Methods

Avastin® was purchased from Roche, Basel, Switzerland, as manufactured by Genentech, Inc., San Francisco, USA (Batch number H0102B01).

Samples of 100 μg protein were applied on immobilized pH 3–10 nonlinear gradient strips. Focusing started at 200 V and the voltage was gradually increased to 8,000 V at 4 V/min and kept constant for a further 3 h (approximately 150,000 Vh totally). Prior to the second dimensional run, strips were equilibrated twice for 15 min with gentle shaking in 10 mL of SDS equilibration buffer (50 mM, pH 8.8, Tris-HCl, 6 M urea, 30% v/v glycerol, 2% w/v SDS, trace of bromophenol blue). DTT (1% w/v) was added at the first incubation for 15 min and 4% (w/v) iodoacetamide instead of DTT at the second incubation step for 15 min. The second-dimensional separation was performed on 10–16% gradient SDS-PAGE. After protein fixation for 12 h in 50% methanol and 10% acetic acid, gels were stained with colloidal Coomassie blue (Novex, San Diego, CA, USA) for 8 h and excess of dye was washed out from the gels with distilled water. Apparent molecular weights were determined by running precision protein standard markers (Bio-Rad Laboratories, Hercules, CA, USA), covering the range of 10–250 kDa and isoelectric points of the immobilized pH gradient strips were from 3-10 [17].

### Sodium hydrosulfite treatment

In order to verify nitration [18], 50 mM sodium hydrosulfite (Na_2_S_2_O_4_) (Sigma, Germany) was added to Avastin® in its original solvent. The reaction mixture was stirred at 23°C for 30 min to convert 3-nitrotyrosine to 3-aminotyrosine. Subsequently buffer was exchanged by 25mM ammonium bicarbonate by Amicon Ultra 10K (Millipore, Billerca, US) 4 times.

### In-gel digestion

**Figure 1:**
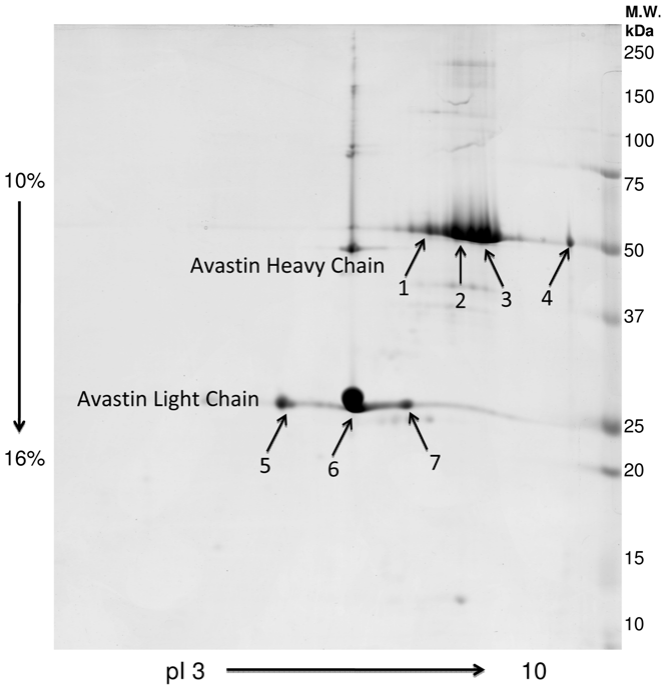
2-DE image of Avastin® is shown providing assignments of identified protein spots.

Selected gel spots were picked for the investigation (Fig. 1). Gel pieces were put into a 1.5 mL tube and washed with 10 mM ammonium bicarbonate and 50% acetonitrile (ACN) in 10 mM ammonium bicarbonate repeatedly. Addition of ACN resulted in gel shrinking and the shrunk gel plugs were then dried in a Speedvac Concentrator (Eppendorf, Germany). Dried gel pieces were re-swollen and in-gel digested with 40 ng/μL trypsin (Promega, Madison, WI, USA) in digestion buffer (consisting of 5 mM octyl β-D-glucopyranoside (OGP) and 10 mM ammonium bicarbonate, pH 7.8) and incubated overnight at 37°C. Digestion with chymotrypsin (Roche Diagnostics), 25 ng/μL was done in 25 mM NH_4_HCO_3_ with 5 mM OGP (pH 7.8) at 30°C for 4 h. Digestion with 40 ng/μL Pepsin (Roche Diagnostics) was done in 0.1M HCL (pH 1) at 37°C for 4 h.

Peptide extractions were performed with 15 μL of 1% formic acid (FA) in 5 mM OGP for 30 min, 15 μL 0.1% FA for 30 min and 15 μL 0.1% FA in 20% ACN for 30 min. The extracted peptides were pooled for nano-LC-ESI-CID/ETD-MS/MS or LTQ orbitrap MS/MS analyses.

### nano-LC-ESI-CID/ETD-MS/MS

The HPLC used was an Ultimate 3000 system (Dionex, Sunnyvale, CA, USA) equipped with a PepMap100 C-18 trap column (300 mm×5mm) and PepMap100 C-18 analytic column (75 mm×150 mm). The gradient was (A 50.1% FA in water, B 50.08% FA in ACN) 4–30% B from 0 to 105 min, 80% B from 105 to 110 min, 4% B from 110 to 125 min. An HCT ultra ETD II (Bruker Daltonics, Bremen, Germany) was used to record peptide spectra over the mass range of m/z 350–1500, and MS/MS spectra in information dependent data acquisition over the mass range of m/z 100–2800. Repeatedly, MS spectra were recorded followed by three data-dependent CID MS/MS spectra and three ETD MS/MS spectra generated from three highest intensity precursor ions. An active exclusion of 0.4 min after two spectra was used to detect low abundant peptides. The voltage between ion spray tip and spray shield was set to 1,500 V. Drying nitrogen gas was heated to 150°C and the flow rate was 10 L/min. The collision energy was set automatically according to the mass and charge state of the peptides chosen for fragmentation. Multiple charged peptides were chosen for MS/MS experiments due to their good fragmentation characteristics. MS/MS spectra were interpreted and peak lists were generated by DataAnalysis 4.0 (Bruker Daltonics).

Raw spectra were processed by Mascot Daemon 2.2.2 (Matrix Science Ltd, London, UK), the search engine was Mascot 2.2.04. Peptide tolerance was set to ±0.2Da, the MS/MS tolerance was set to 0.6 Da. Carbamidomethylcysteine was set as static, oxidation of methionine residues as variable modification. An in-house generated FASTA data base was used for the search containing the sequences of the Avastin® (http://www.pmda.go.jp/english/service/pdf/Abastin-Bevacizumab.pdf), common contaminants and proteolytic enzymes. The data base contained 330 sequences and 110,409 residues. Mascot identifications required at least ion scores greater than 20. PTM searches were also done using the Modiro® software with following parameters: enzyme selected as used with two maximum missing cleavage sites, a peptide mass tolerance of 0.2 Da for peptide tolerance, 0.6 Da for fragment mass tolerance, modification 1 of carbamidomethyl (C) and modification 2 of methionine oxidation. Searches for unknown mass shifts, for amino acid substitutions were carried out and calculation of significance were selected on advanced PTM explorer search strategies. A list of 172 common modifications was selected and added to virtually cleaved and fragmented peptides searched against experimentally obtained MS/MS spectra [19]. Manual inspection of spectra was carried out in the experiments reported.

### LTQ Orbitrap MS

The LC-MS/MS analysis was carried out on an LTQ-Orbitrap Velos ETD mass spectrometer (Thermo Scientific, Waltham, MA, USA). The data acquisition software was XCalibur 2.1.0. The nanospray source of Proxeon (Thermo Scientific, Waltham, MA, USA) was used with the distal coated silica capillaries of New Objective (Woburn, MA, USA). The electrospray voltage was set to 1,500 V. The mass spectrometer was operated in positive ionization mode; the survey scan was performed in the orbitrap, recording a window between 400 and 1,800 m/z. The resolution was set to 60,000 for full MS and the automatic gain control was set to 1,000,000 ions with a maximal acquisition time of 500 ms. The instrument was operated in data-dependent acquisition mode. The minimum MS signal for triggering MS/MS was set to 500, and m/z values triggering fragmentation were put on an exclusion list for 30 s. In all cases one microscan was recorded and a maximum of 20 MS/MS experiments were triggered for the most intense ions from an MS scan. The lock mass option was enabled, and polydimethylcyclosiloxane (protonated (Si(CH_3_)_2_O)_6_; m/z 445.120025 Da) was used for internal recalibration of the mass spectra. CID was applied as fragmentation method with a target value of 1,000 in the linear ion trap, the maximal acquisition time was 100 ms, the collision energy was set to 35%, and the Q value to 0.25. An activation time of 10 ms was applied. Singly charged ions and ions with unassigned charge states were not fragmented. Helium was used as collision gas.

Raw spectra were processed by Mascot Daemon 2.2.2 (Matrix Science Ltd, London, UK), the search engine was Mascot 2.2.04. Peptide tolerance was set to ±2ppm, the MS/MS tolerance was set to 0.8 Da. Otherwise data mining was carried out as given above.

## Results and Discussion

As shown in Fig. 1 the commercially available protein drug Avastin® was presenting with several spots. Heavy and light chain were clearly separated and heavy chain protein showed tracking while the light chain showed three major spots. Seven spots were picked and sequence coverages, enzyme that generated matching peptides, peptide sequences, ion scores and mass errors that were less than 0.2 Da are provided in Table S1 and Figure S1. Only spots 2,3,6 and 7 were presenting with tyrosine nitrations. The proposed multiple protein modifications (PMs) rather than sequence conflicts or amino acid exchanges observed in the individual spots resulting from HCT analysis respecting CID and ETD fragmentations may have been responsible for horizontal and vertical electrophoretic shifts as given in Table S2A.

LTQ-orbitrap analyses were used to find additional PMs in some Avastin® spots, and showed the presence of PMs and the results are demonstrated in Table S2B. PMs observed by both methods are presented in Table 1. Results on different modifications of avastin® expression forms are assigned to spot numbers.

**Table 1:** The modifications verified by HCT and Orbitrap.

Spot	Modifications from MODIRO®
2	Deamidation: R87, N321, N390
	Dihydroxy: Y284
	Methylation: T266
	Oxidation: M34, M83
4	Deamidation: N321
	Dihydroxy: Y284
	Hydroxylation: D73
	Methylation: E6
	Oxidation: M34, M83
6	Amino: Y192
	Deamidation: R142, Q199
	Methylation: S121, D122, E123
	Nitro: Y192
	Oxidation: M4
7	Deamidation: Q199
	Dihydroxy: Y36, T197
	Hydroxylation: K39
	Methylation: E123, E195
	Oxidation: M4

Table 2 shows tyrosine nitrations shown by ion trap using CID but not by ETD fragmentation as well as using LTQ orbitrap.

**Table 2:** Identified nitrotyrosine or aminotyrosine modifications in Avastin by HCT and Orbitrap: the software proposes possible modifications on the peptide and subsequently assigns them to “measured m/z”. The theoretical m/z indicates the peptide mass with expected modifications.

Chain	Spot	Enzyme	m/z meas.^d^	m/z theor. d	Error d	z	Peptide	Pos.	Score	Sig.	Modification
AHC^a^ (HCT)	2	Chymo	956.47	956.4691	0.0009	2	L.GC^CAMe^LVKDY^Amino^FPEPVTVSW.N	149-164	229	99.9	Amino (Y155)
	2	Try	845.12	845.0727	0.0473	3	K.GLEWVGWINTY^Amino^TGEPTYAADFK.R	44-65	265	88.5	Amino (Y54)
	3	Try	846.85	846.9101	-0.0601	2	K.FNWY^Amino^VDGVEVHNAK.T	281-294	256	100	Amino (Y284)
ALC^b^ (HCT)	6	Try	945.84	945.9726	-0.1325	2	K.VY^Amino^AC^CAMe^EVTHQGLSSPVTK.S	191-207	263	99.9	Amino (Y192)^c^
	6	Try	946.36	946.4435	-0.0835	3	K.ADY^Nitro^EKHKVY^Nitro^AC^CAMe^EVTHQGLSSPVTK.S	184-207	252	88.5	Nitro (Y186, Y192)^c^
	7	Try	945.89	945.9726	-0.0825	2	K.VY^Amino^AC^CAMe^EVTHQGLSSPVTK.S	191-207	249	99.4	*Amino (Y192)*
AHC^a^ (Orbitrap)	2	Try	1129.983	1129.9833	-0.0003	2	R.LSC^CAMe^AASGY^Nitro^TFTNYGM^Ox^NWVR.Q	20 - 38	292	100	Nitro (Y27)
	2	Try	672.8138	672.8143	-0.0005	2	K.STAY^Nitro^LQM^Ox^NSLR.A	77 - 87	327	100	Nitro (Y80)
	2	Try	975.4663	975.4673	-0.001	2	R.EPQVY^Nitro^TLPPSREEMTK.N	351 - 366	229	99.9	Nitro (Y355)
	2	Try	959.9564	959.9571	-0.0007	2	K.TTPPVLDSDGSFFLY^Nitro^SK.L	399 - 415	288	100	Nitro (Y413)
ALC^b^ (Orbitrap)	6	Try	945.9722	945.9726	0.0015	2	K.VY^Amino^AC^CAMe^EVTHQGLSSPVTK.S	191 - 207	242	100	Amino (Y192)^c^
	6	Try	949.4517	949.452	-0.0003	3	R.VTITC^CAMe^SASQDISNYLNWY^Nitro^QQKPGK.A	19 - 42	280	100	Nitro (Y36)
	6	Try	904.47	904.4705	-0.0005	2	K.VLIY^Nitro^FTSSLHSGVPSR.F	46 - 61	407	100	Nitro (Y49)
	6	Try	921.9441	921.9438	0.0003	2	K.SGTASVVC^CAMe^LLNNFY^Nitro^PR.E	127 - 142	389	100	Nitro (Y140)
	6	Try	774.3751	774.3754	-0.0003	2	K.DSTY^Nitro^SLSSTLTLSK.A	170 - 183	376	100	Nitro (Y173)
	6	Try	960.9589	960.9596	-0.0009	3	K.VY^Nitro^AC^CAMe^EVTHQGLSSPVTK.S	191 - 207	286	100	Nitro (Y192)^c^
	7	Try	603.3159	603.3161	-0.0002	3	K.VLIY^Nitro^FTSSLHSGVPSR.F	46 - 61	369	99.4	Nitro (Y49)
	7	Try	774.3754	774.3754	0	2	K.DSTY^Nitro^SLSSTLTLSK.A	170 - 183	306	100	Nitro (Y173)
	7	Try	640.9743	640.9755	-0.0012	3	K.VY^Nitro^AC^CAMe^EVTHQGLSSPVTK.S	191 - 207	228	98.9	*Nitro (Y192)*

a Avastin Heavy chain

b Avastin Light chain

c Both nitrotyrosine and aminotyrosine modifications are identified at the same Y192 site of Spot 6.

d given in Da

On the light chain as well as on the heavy chain of the antibody (Avastin®) LTQ identified this PM. HCT analysis demonstrated two tyrosine nitrations on the light chain and three at the heavy chain. LTQ orbitrap detected five tyrosine nitrations on the light chain and four on the heavy chain. Nitration was observed as nitration or as aminotyrosine [20]. Both MS/MS methods detected nitration of Y192 expressed by nitrotyrosine and aminotyrosine identification.

To support the identification of nitrotyrosine, Avastin® was reduced by sodium hydrosulfite and indeed, aminotyrosine on Y192 was detected (Figure S2), which does not rule out that the other modifications are not valid: results from different methodologies depend on fragmentation and formation of corresponding ions generated. Misidentifications of tyrosine nitration by a series of factors was ruled out [21].

Multiple nitrations of the humanised antibody Avastin® may have chemical and biological consequences: potential changes of Avastin immunochemistry may include antigenic changes and nitration has been repeatedly shown to modify this immunological property and was even leading to autoimmune phenomena [22]. The underlying cause of nitrations cannot be addressed but chemical modification by manufacturing is as plausible as nitration that occurred in vivo during primary generation of the antibody as a posttranslational modification [23]. Details from manufacturing and production are not available from the supplier or the corresponding patent (6,054,297 US patent, 2000).

Antibodies against nitrotyrosine have been observed [24] and it is known that nitrated peptides and proteins are eliciting antibody formation [25]-[27]. Moreover, antibodies against nitrated alpha synuclein produced an inflammatory response in mice that led to degeneration of dopaminergic neurons [28]. One may also suggest that nitration not only leads to autoantibody formation but also to conformational changes of the nitrated protein [29] that in turn may change antigenic properties and also protein-protein interactions with unknown functional consequences. Apart from nitration-induced antigenic changes of epitopes, nitration is known to impair function of proteins including Mn superoxide dismutase [30], [31].

A major finding indicating pathogenetic or toxic properties of nitrated proteins was presented recently: assembly of alpha-synuclein and fibrinogen were deteriorated by nitration of only a small fraction of proteins [25], [32]. As for fibrinogen, nitration is accelerating the rate of fibrin clot formation [33]. It cannot be ruled out, therefore, that nitration of Avastin® may lead to side effects based upon probable disturbed protein-protein interactions as aggregations that in turn may lead to a series of complications as listed in the drug information sheet.

As to the underlying cause of tyrosine nitration it remains open, whether it can be considered as a post-translational modification of the immunoglobulin Avastin® or technical in nature, or both [34]-[38].

Taken together, it remains unclear if the observed nitrations and other PMs detected on Avastin® may be responsible for different biological or pharmacotoxicological properties and effects. Quantification of nitrations and their presence in several batches of the product has to be taken into considerations on probable toxicity as well, but corresponding *in vivo* analyses based upon these findings should be carried out by the manufacturers to probably save this protein drug that may have strong potential in tumor therapy.

## Supporting Information

Figure S1 **Sequence information of Avastin® is shown.** - identified by A) HCT and B) Orbitrap.
(PDF)

Figure S2 **Spectra of aminotyrosine and nitrotyrosine modifications.**
(PDF)

Table S1 **MS/MS results of from Avastin (MASCOT) identified by HCT (7 spots) and by Orbitrap (4 spots).**
(PDF)

Table S2 **The modifications revealed by the Modiro search engine from HCT and Orbitrap data.**
(PDF)
