# Supplementary material for: Proteins from Avastin® (bevacizumab) Show Tyrosine Nitrations for which the Consequences Are Completely Unclear
Source: PLoS One. 2012 Apr 16;7(4):e34511. doi: 10.1371/journal.pone.0034511 (PMC3327692; doi:10.1371/journal.pone.0034511)
Supplement: Figure S1 — Sequence information of Avastin® is shown. - identified by A) HCT and B) Orbitrap. (PDF) [file pone.0034511.s001.pdf]

Figure S1

Sequence information of Avastin® identified by A) HCT and B) Orbitrap is shown

A

Avastin heavy chain (sequence coverage 98%)

Spot 1 (sequence coverage 64%) identified by HCT

1 **EVQLVESGGG LVQPGGSLRL SCAASGYTFT NYGMNWVR**QA PGKGLEWVGW
51 **INTYTGEPTY AADFK**RRFTF SLDTSK**STAY LQMNSLRAED TAVYYCAK**YP
101 HYYGSSHWYF DVWGQGTLVT VSSASTK**GPS VFPLAPSSKS TSGGTAALGC**
151 **LVKDYFPEPV TVSWNSGALT S**GVHTFPAVL QSSGLY**SLSS VVTVPSSSLG**
201 **TQTYICNVN**H KPSNTKVDKK VEPKSCDK**TH TCPPCAPEL LGGPSVFLFP**
251 **PKPK**DTLMIS R**TPEVTCVVV DVSHEDPEVK FNWYVDGVEV HNAK**TKPREE
301 QYNSTYR**VVS VLTVLHQDWL NGK**EYKCKVS NKALPAPIEK TISKAKGQPR
351 **EPQVYTLPPS R**EEMTK**NQVS LTCLVKGFYP SDIAVEWESN GQPENNYKTT**
401 **PPVLDSDGSF FLYSK**LTVDK SRWQQGNVF**S CSVMHEALHN HYTQK**SLSLS
451 PGK

Spot 2 (sequence coverage 96%) identified by HCT

1 **EVQLVESGGG LVQPGGSLRL SCAASGYTFT NYGMNWVRQA PGKGLEWVGW**
51 **INTYTGEPTY AADFK**R**RFTF SLDTSKSTAY LQMNSLRAED TAVYYCAKYP**
101 **HYYGSSHWYF DVWGQGTLVT VSSASTKGPS VFPLAPSSKS TSGGTAALGC**
151 **LVKDYFPEPV TVSWNSGALT SGVHTFPAVL QSSGLYSLSS VVTVPSSSLG**
201 **TQTYICNVNH KPSNTKVDKK VEPKSCDKTH TCPPCAPEL LGGPSVFLFP**
251 **PKPKDTLMIS RTPEVTCVVV DVSHEDPEVK FNWYVDGVEV HNAK**TKPREE
301 QYNSTY**RVVS VLTVLHQDWL NGKEYKCKVS NKALPAPIEK TISKAKGQPR**
351 **EPQVYTLPPS R**EEMT**KNQVS LTCLVKGFYP SDIAVEWESN GQPENNYKTT**
401 **PPVLDSDGSF FLYSKLTVDK SRWQQGNVFS CSVMHEALHN HYTQKSLSLS**
451 **PG**K

Spot 3 (sequence coverage 95%) identified by HCT

1 **EVQLVESGGG LVQPGGSLRL SCAASGYTFT NYGMNWVRQA PGKGLEWVGW**
51 **INTYTGEPTY AADFK**R**RFTF SLDTSKSTAY LQMNSLRAED TAVYYCAKYP**
101 **HYYGSS**HW**YF DVWGQGTLVT VSSASTKGPS VFPLAPSSKS TSGGTAALGC**
151 **LVKDYFPEPV TVSWNSGALT SGVHTFPAVL QSSGLY**SLSS **VVTVPSSSLG**
201 **TQTYICNVNH KPSNTKVDKK VEPKSCDKTH TCPPCAPEL LGGPSVFLFP**
251 **PKPKDTLMIS RTPEVTCVVV DVSHEDPEVK FNWYVDGVEV HNAK**TKPREE
301 QYNSTY**RVVS VLTVLHQDWL NGKEYKCKVS NKALPAPIEK TISKAKGQPR**
351 **EPQVYTLPPS R**EEMT**KNQVS LTCLVKGFYP SDIAVEWESN GQPENNYKTT**
401 **PPVLDSDGSF FLYSKLTVDK SRWQQGNVFS CSVMHEALHN HYTQKSLSLS**
451 **PG**K

Spot 4 (sequence coverage 83%) identified by HCT

1 **EVQLVESGGG LVQPGGSLRL SCAASGYTFT NYGMNWVRQA PGKGLEWVGW**
51 **INTYTGEPTY AADFK**R**RFTF SLDTSKSTAY LQMNSLRAED TAVYYCAK**YP
101 HYYGSSHWYF DVWGQGTLVT VSSASTK**GPS VFPLAPSSKS TSGGTAALGC**
151 **LVKDYFPEPV TVSWNSGALT SGVHTFPAVL QSSGLYSLSS VVTVPSSSLG**
201 **TQTYICNVNH KPSNTK**VDKK VEPKSCDK**TH TCPPCAPEL LGGPSVFLFP**
251 **PKPKDTLMIS RTPEVTCVVV DVSHEDPEVK FNWYVDGVEV HNAK**TKPREE
301 QYNSTYR**VVS VLTVLHQDWL NGK**EYKCKVS NKALPAPIEK TISKAKGQPR
351 **EPQVYTLPPS REEMTKNQVS LTCLVKGFYP SDIAVEWESN GQPENNYKTT**
401 **PPVLDSDGSF FLYSKLTVDK SRWQQGNVFS CSVMHEALHN HYTQKSLSLS**
451 **PG**K

Avastin light chain (sequence coverage 100%)

Spot 5 (sequence coverage 100%) identified by HCT

1 **DIQMTQSPSS LSASVGDRVT ITCSASQDIS NYLNWYQQKP GKAPKVLIYF**
51 **TSSLHSGVPS RFSGSGSGTD FTLTISSLQP EDFATYYCQQ YSTVPWTFGQ**
101 **GTKVEIKRTV AAPSVFIFPP SDEQLKSGTA SVVCLLNNFY PREAKVQWKV**
151 **DNALQSGNSQ ESVTEQDSKD STYSLSSTLT LSKADYEKHK VYACEVTHQG**
201 **LSSPVTKSFN RGEC**

Spot 6 (sequence coverage 88%) identified by HCT

1 **DIQMTQSPSS LSASVGDRVT ITCSASQDIS NYLNWYQQKP GKAPKVLIYF**
51 **TSSLHSGVPS RF**SGSGSGTD FTLTISSLQP EDFATY**YCQQ YSTVPWTFGQ**
101 **GTKVEIKRTV AAPSVFIFPP SDEQLKSGTA SVVCLLNNFY PREAKVQWKV**
151 **DNALQSGNSQ ESVTEQDSKD STYSLSSTLT LSKADYEKHK VYACEVTHQG**
201 **LSSPVTKSFN RGEC**

Spot 7 (sequence coverage 78%) identified by HCT

1 **DIQMTQSPSS LSASVGDRVT ITCSASQDIS NYLNWYQQKP GK**APK**VLIYF**
51 **TSSLHSGVPS R**FSGSGSGTD FTLTISSLQP EDFATYY**CQQ YSTVPWTFGQ**
101 **GTK**VEIK**RTV AAPSVFIFPP SDEQLKSGTA SVVCLLNNFY PR**EAKVQWKV
151 **DNALQSGNSQ ESVTEQDSKD STYSLSSTLT LSK**ADYEKHK **VYACEVTHQG**
201 **LSSPVTK**SFN RGEC

B

Avastin heavy chain (sequence coverage 97%)

Spot 2 (sequence coverage 76%) identified by Orbitrap

1 EVQLVESGGG LVQPGGSLRL SCAASGYTFT NYGMNWVRQA PGKGLEWVGW
51 INTYTGEPTY AADFKRRFTF SLDTSKSTAY LQMNSLRAED TAVYYCAKYP
101 HYYGSSHWYF DVWGQGTLVT VSSASTKGPS VFPLAPSSKS TSGGTAALGC
151 LVKDYFPEPV TVSWNSGALT SGVHTFPAVL QSSGLYSLSS VVTVPSSSLG
201 TQTYICNVNH KPSNTKVDKK VEPKSCDKTH TCPPCPAPEL LGGPSVFLFP
251 PKPKDTLMIS RTPEVTCVVV DVSHEDPEVK FNWYVDGVEV HNAKTKPREE
301 QYNSTYRVVS VLTVLHQDWL NGKEYKCKVS NKALPAPIEK TISKAKGQPR
351 EPQVYTLPPS REEMTKNQVS LTCLVKGFYP SDIAVEWESN GQPENNYKTT
401 PPVLDSDGSF FLYSKLTVDK SRWQQGNVFS CSVMHEALHN HYTQKSLSLS
451 PGK

Spot 4 (sequence coverage 83%) identified by Orbitrap

1 EVQLVESGGG LVQPGGSLRL SCAASGYTFT NYGMNWVRQA PGKGLEWVGW
51 INTYTGEPTY AADFKRRFTF SLDTSKSTAY LQMNSLRAED TAVYYCAKYP
101 HYYGSSHWYF DVWGQGTLVT VSSASTKGPS VFPLAPSSKS TSGGTAALGC
151 LVKDYFPEPV TVSWNSGALT SGVHTFPAVL QSSGLYSLSS VVTVPSSSLG
201 TQTYICNVNH KPSNTKVDKK VEPKSCDKTH TCPPCPAPEL LGGPSVFLFP
251 PKPKDTLMIS RTPEVTCVVV DVSHEDPEVK FNWYVDGVEV HNAKTKPREE
301 QYNSTYRVVS VLTVLHQDWL NGKEYKCKVS NKALPAPIEK TISKAKGQPR
351 EPQVYTLPPS REEMTKNQVS LTCLVKGFYP SDIAVEWESN GQPENNYKTT
401 PPVLDSDGSF FLYSKLTVDK SRWQQGNVFS CSVMHEALHN HYTQKSLSLS
451 PGK

Avastin heavy chain (sequence coverage 100%)

Spot 6 (sequence coverage 100%) identified by Orbitrap

1 DIQMTQSPSS LSASVGDRVT ITCSASQDIS NYLNWYQQKP GKAPKVLIYF
51 TSSLHSGVPS RFSGSGSGTD FTLTISSLQP EDFATYYCQQ YSTVPWTFGQ
101 GTKVEIKRTV AAPSVFIFPP SDEQLKSGTA SVVCLLNNFY PREAKVQWKV
151 DNALQSGNSQ ESVTEQDSKD STYSLSSTLT LSKADYEKHK VYACEVTHQG
201 LSSPVTKSFN RGEC

Spot 7 (sequence coverage 95%) identified by Orbitrap

1 DIQMTQSPSS LSASVGDRVT ITCSASQDIS NYLNWYQQKP GKAPKVLIYF
51 TSSLHSGVPS RFSGSGSGTD FTLTISSLQP EDFATYYCQQ YSTVPWTFGQ
101 GTKVEIKRTV AAPSVFIFPP SDEQLKSGTA SVVCLLNNFY PREAKVQWKV
151 DNALQSGNSQ ESVTEQDSKD STYSLSSTLT LSKADYEKHK VYACEVTHQG
201 LSSPVTKSFN RGEC
