# Supplementary material for: Proteins from Avastin® (bevacizumab) Show Tyrosine Nitrations for which the Consequences Are Completely Unclear
Source: PLoS One. 2012 Apr 16;7(4):e34511. doi: 10.1371/journal.pone.0034511 (PMC3327692; doi:10.1371/journal.pone.0034511)

Figure S2

Nitro-Y192 in Avastin® light chain identified by Orbitrap

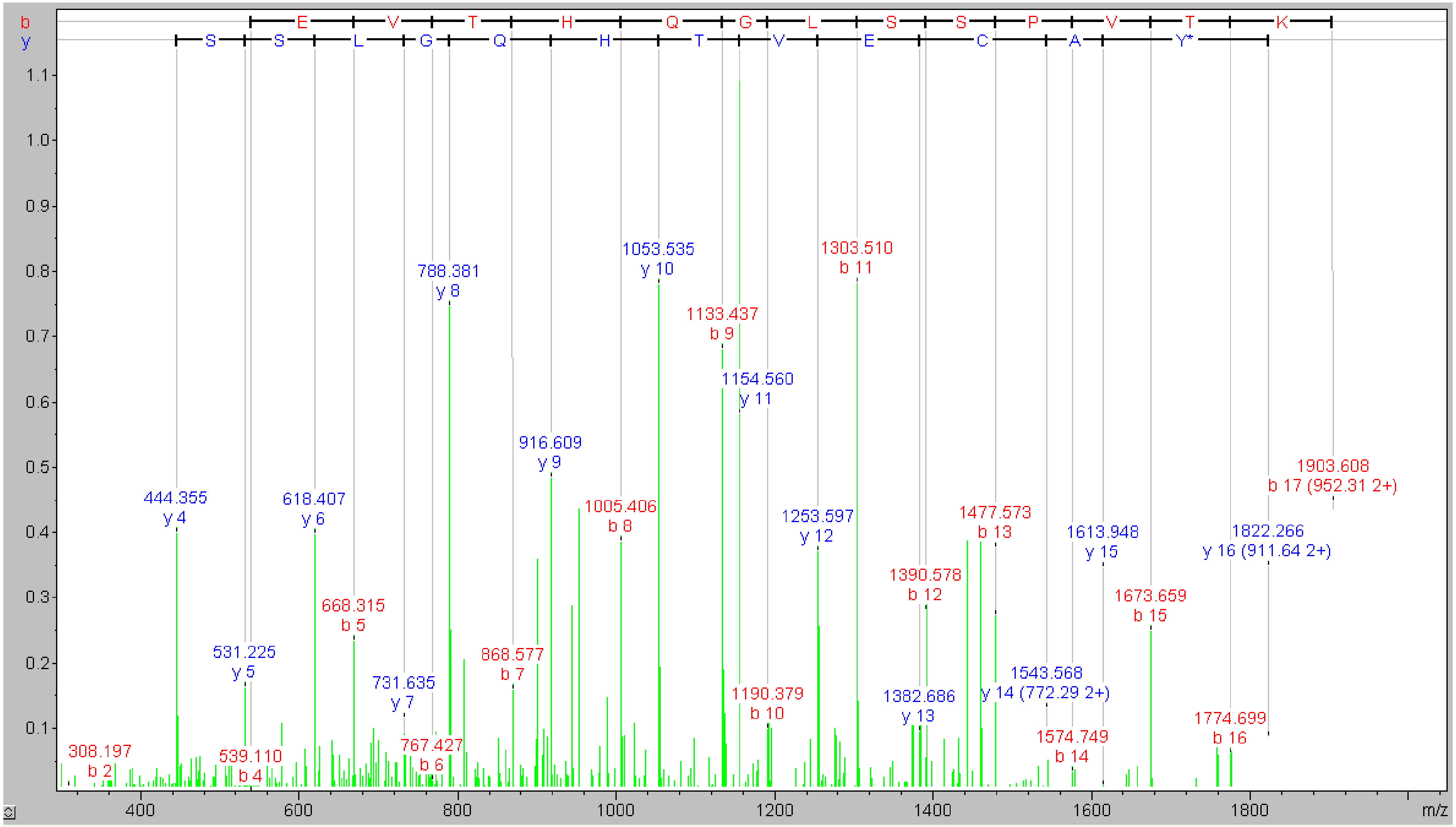

K.VY^{Nitro}AC^{CAMe}EVTHQGLSSPVTK.S

Amino-Y192 in Avastin® light chain identified by Orbitrap

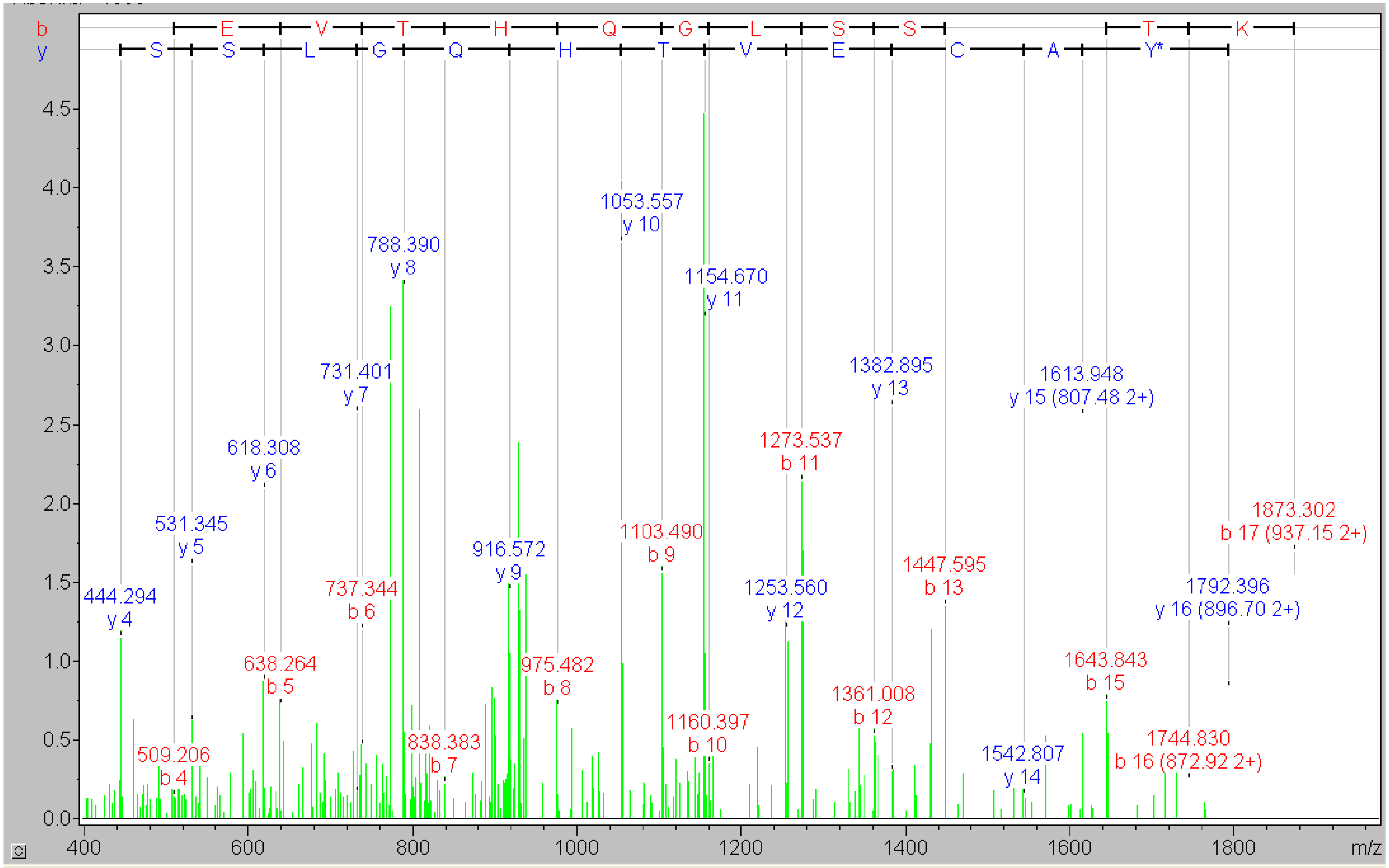

K. VY^{Amino}AC^{CAMe}EVTHQGLSSPVTK.S

Amino-Y192 in Avastin® light chain identified by HCT

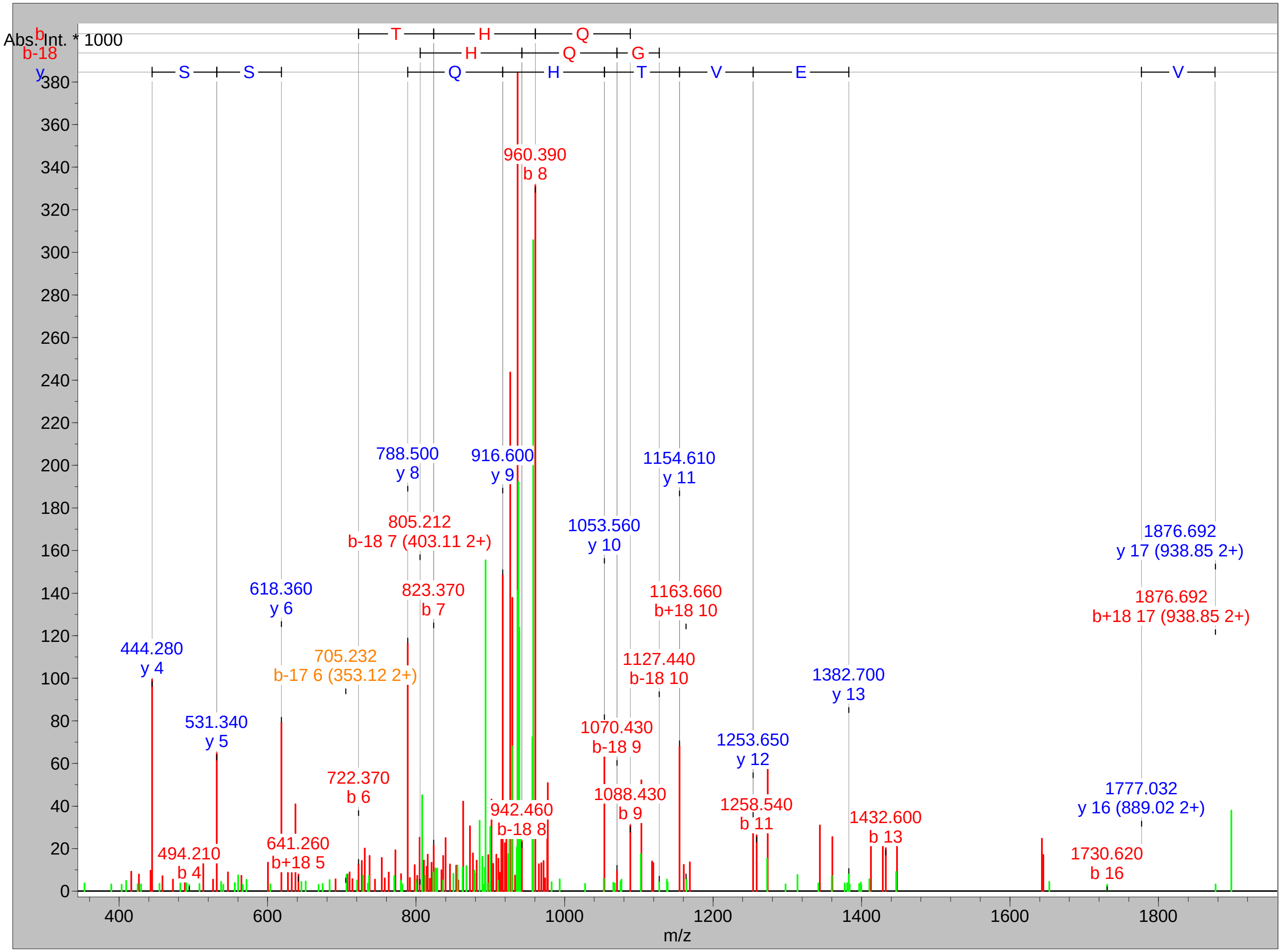

K.VY^{Amino}AC^{CAMe}EVTHQGLSSPVTK.S

Nitro-Y192 in Avastin® light chain identified by HCT

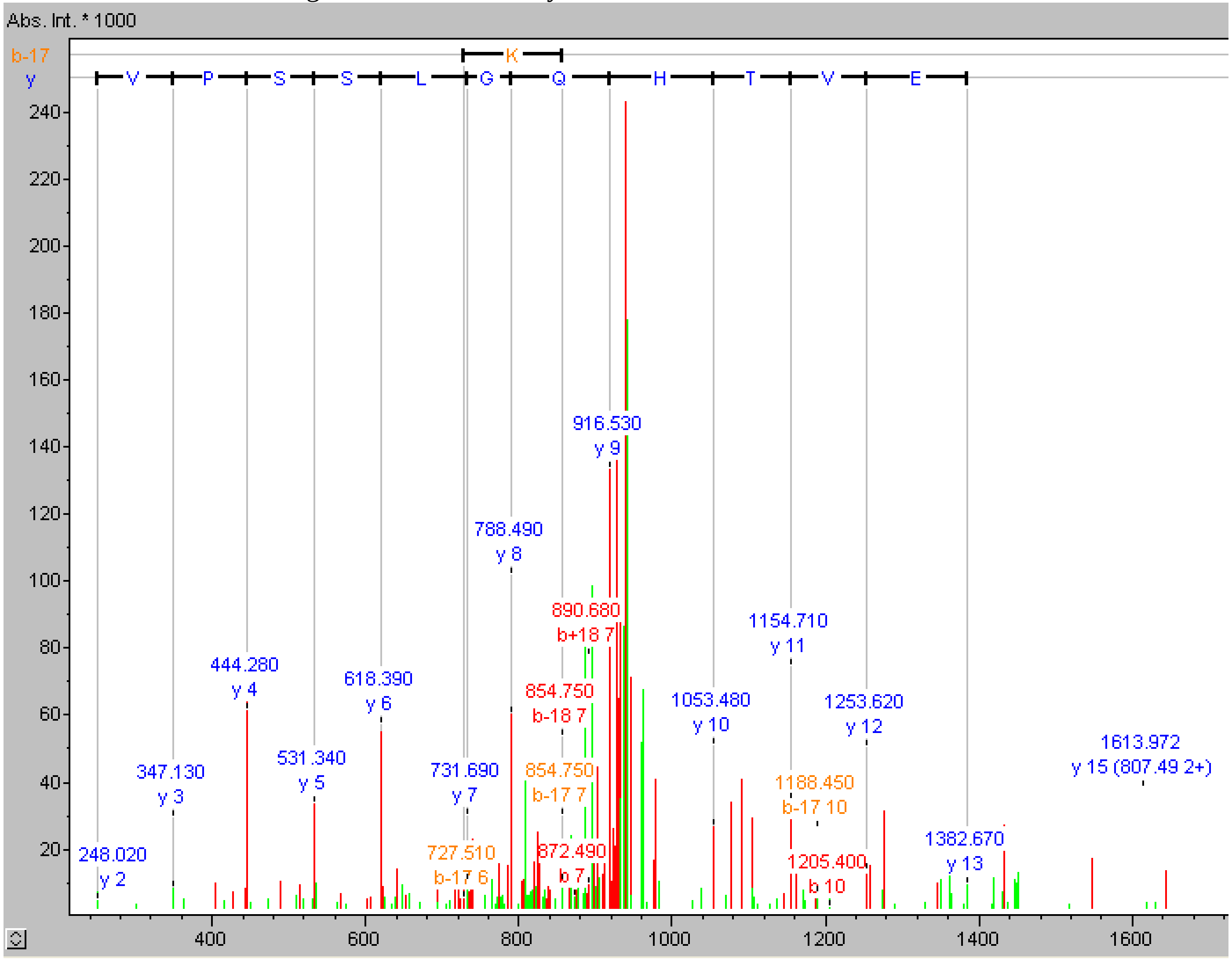

K.ADY^{Nitro}EKHKVY^{Nitro}AC^{CAMe}EVTHQGLSSPVTK.S

Conversion of nitrotyrosine to aminotyrosine by reduction with $Na_2S_2O_4$ for light chain Y192.

(1) without treatment, (2) following $Na_2S_2O_4$ treatment

(1) K.VY^{Nitro}AC^{CAMe}EVTHQGLSSPVTK.S

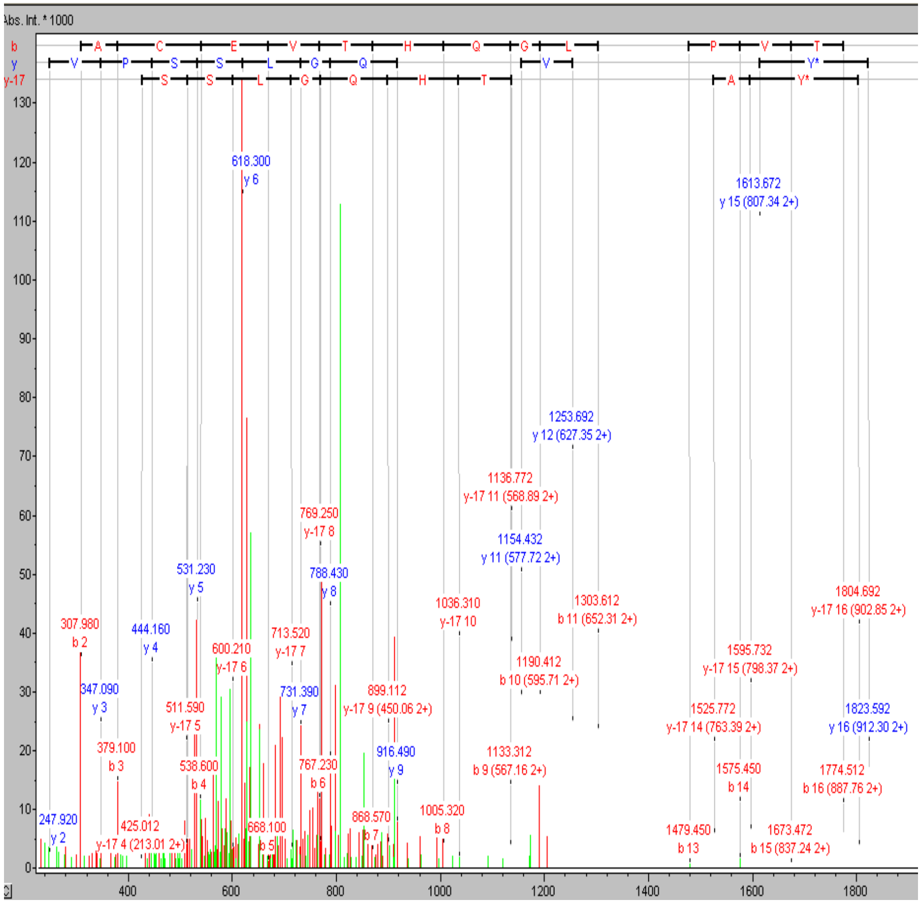

(2) K.VY^{Amino}AC^{CAMe}EVTHQGLSSPVTK.S

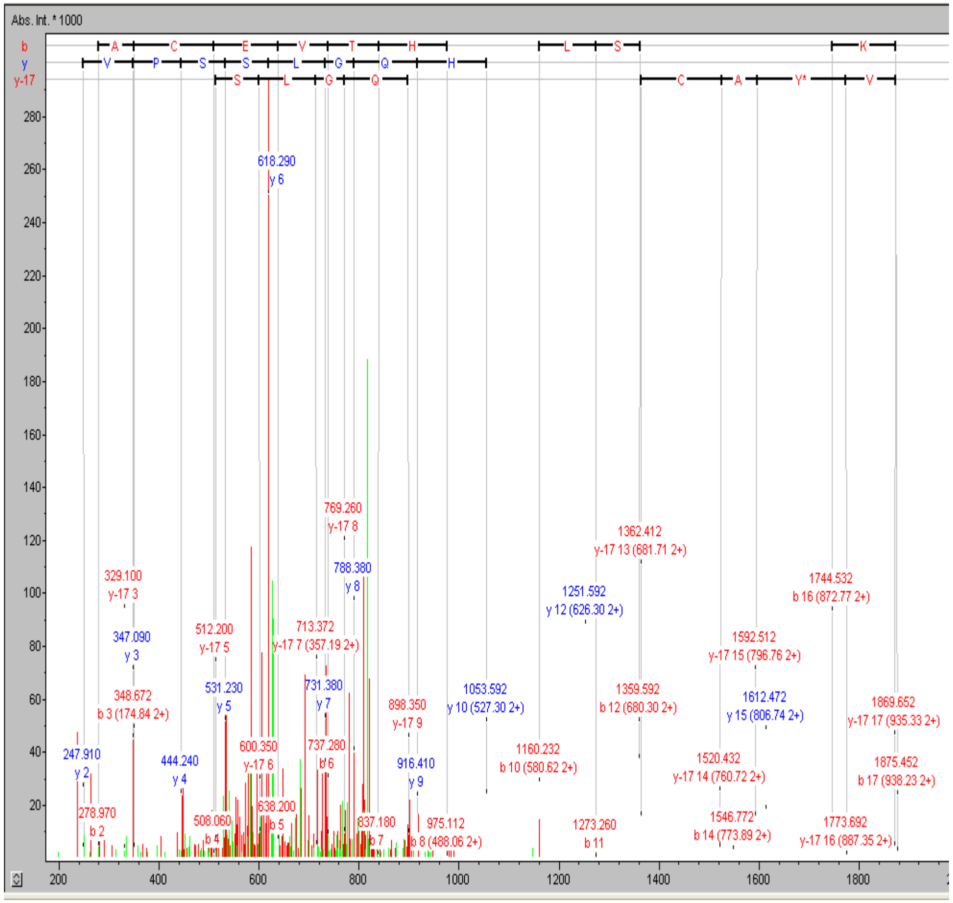

Supplement: Figure S2 — Spectra of aminotyrosine and nitrotyrosine modifications. (PDF) [file pone.0034511.s002.pdf]
