# Supplementary material for: Proteins from Avastin® (bevacizumab) Show Tyrosine Nitrations for which the Consequences Are Completely Unclear
Source: PLoS One. 2012 Apr 16;7(4):e34511. doi: 10.1371/journal.pone.0034511 (PMC3327692; doi:10.1371/journal.pone.0034511)
Supplement: Table S1 — MS/MS results of from Avastin (MASCOT) identified by HCT (7 spots) and by Orbitrap (4 spots). (PDF) [file pone.0034511.s003.pdf]

Table S1A

MS/MS results of 7 spots from Avastin (MASCOT) Identified by HCT

Avastin was separated by 2-DE and identified by nano-LC-ESI-CID/ETD-MS/MS using the MASCOT search engine.

Spot	Coverage	Identified Peptide (enzyme/ion score/mass error)
1	64%	1 -.EVQLVESGGGLVQPGGSLR.L 19 (Try/122/-0.1042)<br>3 V.QLVESGGGLVQPGGSLR.L 19 (Try/101/0.0307)<br>20 R.LSCAASGYTF.T 29 (Try/30/-0.0457)<br>20 R.LSCAASGYTFTNYGM.N 34 (Try/79/0.0857)<br>20 R.LSCAASGYTFTNYGM*N.W Oxidation(M) 35 (Try/69/0.0151)<br>20 R.LSCAASGYTFTNYGMNWVR.Q 38 (Try/108/0.0564)<br>28 Y.TFTNYGMNWVR.Q 38 (Try/37/0.1675)<br>51 W.INTYTGEPTYAADFK.R 65 (Try/73/-0.1062)<br>53 N.TYTGEPTYAADFK.R 65 (Try/69/0.0056)<br>77 K.STAYLQMNSLR.A 87 (Try/39/0.0311)<br>77 K.STAYLQM*NSLR.A Oxidation(M) 87 (Try/63/0.077)<br>80 A.YLQMNSLR.A 87 (Try/30/0.1719)<br>88 R.AEDTAVYYCAK.Y 98 (Try/79/0.0829)<br>128 K.GPSVFPLAPSSK.S 139 (Try/44/-0.0045)<br>129 G.PSVFPLAPSSK.S 139 (Try/34/0.1217)<br>140 K.STSGGTAALGCLVK.D 153 (Try/63/0.0663)<br>142 T.SGGTAALGCLVK.D 153 (Try/56/0.1502)<br>154 K.DYFPEPVTVSWN.S 165 (Try/64/0.1589)<br>154 K.DYFPEPVTVSWNSGAL.T 169 (Try/41/-0.0076)<br>154 K.DYFPEPVTVSWNSGALTS.G 171 (Try/62/-0.0731)<br>187 Y.SLSSVVTVPSSSLGTQTY.I 204 (Try/38/0.0799)<br>189 L.SSVVTVPSSSLGTQTY.I 204 (Try/41/0.0204)<br>189 L.SSVVTVPSSSLGTQTYICNVN.H 209 (Try/54/-0.0827)<br>190 S.SVVTVPSSSLGTQTYICNVN.H 209 (Try/78/-0.0301)<br>229 K.THTCPPCPAPELLGGPS.V 245 (Try/66/0.0887)<br>229 K.THTCPPCPAPELLGGPSVF.L 247 (Try/36/-0.021)<br>238 A.PELLGGPSVFLFPPKPK.D 254 (Try/57/-0.012)<br>262 R.TPEVTCVVVDVSHED.P 276 (Try/70/-0.0124)<br>262 R.TPEVTCVVVDVSHEDPEVK.F 280 (Try/69/0.1366)<br>281 K.FNWYVDGVEVHNAK.T 294 (Try/92/0.0217)<br>308 R.VVSVLTVLHQDWLNGK.E 323 (Try/69/0.0032)<br>351 R.EPQVYTLPPSR.E 361 (Try/50/0.0428)<br>352 E.PQVYTLPPSR.E 361 (Try/68/0.0596)<br>367 K.NQVSLTCLVK.G 376 (Try/43/-0.0621)<br>377 K.GFYPSDIAVEWESNGQPENNYK.T 398 (Try/37/0.1155)<br>399 K.TTPPVLDSDGSFFLY.S 413 (Try/62/0.0637)<br>401 T.PPVLDSDGSFFLYSK.L 415 (Try/103/0.0856)<br>430 F.SCSVMHEALHNHYTQK.S 445 (Try/53/-0.0259)
2	96%	1 -.EVQLVESGGGLVQPGGSLRLSCAASGY.T 27 (Chy/35/0.019)

		1 -.EVQLVESGGGLVQPGGSLR.L 19 (Try/94/-0.1454)<br>4 Q.LVESGGGLVQPGGSLR.L 19 (Try/41/0.011)<br>4 Q.LVESGGGLVQPGGS.L 17 (Pep/40/-0.0176)<br>4 Q.LVESGGGLVQPGGSL.R 18 (Pep/39/0.1023)<br>5 L.VESGGGLVQPGGSLRL.S 20 (Pep/44/0.1566)<br>14 Q.PGGSL.R 18 (ProK/32/0.0536)<br>20 R.LSCAASGYTFTNYGMNWVR.Q 38 (Chy/104/-0.1228)<br>20 R.LSCAASGYTFTNYGMNWVR.Q 38 (Try/129/-0.1398)<br>20 R.LSCAASGYTFTNYGM*NWVR.Q Oxidation(M) 38 (Try/98/-0.1966)<br>21 L.SCAASGY.T 27 (Chy/31/0.0629)<br>23 C.AASGYTFTNYGMNWVR.Q 38 (Try/72/0.0049)<br>25 A.SGYTFTNYGMNWVR.Q 38 (Try/44/0.0155)<br>28 Y.TFTNYGMNWVR.Q 38 (Try/57/0.0823)<br>36 N.WVRQAPGKGLE.W 46 (Pep/44/0.0977)<br>36 N.WVRQAPGKGLEW.V 47 (Pep/42/0.0867)<br>36 N.WVRQAPGKGLEWVG.W 49 (Pep/45/0.1176)<br>44 K.GLEWVGWINTYTGEPTYAADFK.R 65 (Chy/64/-0.0982)<br>44 K.GLEWVGWINTY.T 54 (Try/67/0.0785)<br>44 K.GLEWVGWINTYTGEPT.Y 59 (Try/38/-0.0413)<br>44 K.GLEWVGWINTYTGEPTYAADFK.R 65 (Try/142/-0.172)<br>53 N.TYTGEPTYAADFK.R 65 (Try/37/0.1066)<br>55 Y.TGEPTYAADF.K 64 (Chy/31/-0.0055)<br>55 Y.TGEPTYAADFK.R 65 (Try/33/0.0288)<br>56 T.GEPTYAADFK.R 65 (Try/40/-0.1127)<br>67 R.RFTFSLDTSK.S 76 (Try/42/0.097)<br>71 F.SLDTSKSTAY.L 80 (Chy/43/0.18)<br>73 L.DTSKSTAY.L 80 (Chy/40/0.0273)<br>74 D.TSKSTAY.L 80 (Chy/37/0.0948)<br>77 K.STAYLQMNSLR.A 87 (Try/46/0.0563)<br>77 K.STAYLQM*NSLR.A Oxidation(M) 87 (Try/58/0.086)<br>81 Y.LQMNSLRAEDTAVY.Y 94 (Chy/42/0.0267)<br>87 L.RAEDTAVY.Y 94 (Pep/48/-0.0209)<br>88 R.AEDTAVYYCAK.Y 98 (Try/80/0.1271)<br>94 V.YYCAKYPHY.Y 102 (Pep/34/0.0194)<br>94 V.YYCAKYPHYYGSS.H 106 (Pep/37/-0.0309)<br>95 Y.YCAKYPHYYGSS.H 106 (Pep/42/0.0653)<br>95 Y.YCAKYPHYYGSSHW.Y 108 (Pep/40/-0.0978)<br>109 W.YFDVWGQGTL.V 118 (Chy/35/-0.0471)<br>119 L.VTVSSASTKGPSVF.P 132 (Pep/55/0.1004)<br>119 L.VTVSSASTKGPSVFPLAPSSKSTSGGTAAL.G 148 (Pep/41/0.0643)<br>121 T.VSSASTKGPSVFPLAPSSKSTSGGTAAL.G 148 (Pep/39/-0.117)<br>122 V.SSASTKGPSVFPLAPSSKSTSGGTAAL.G 148 (Chy/38/0.0429)<br>125 A.STKGPSVFPLAPS.S 137 (Pep/31/0.1378)<br>127 T.KGPSVFPLAPS.S 137 (Pep/34/0.02)<br>128 K.GPSVFPLAPSSK.S 139 (Try/49/-0.0171)<br>133 F.PLAPSSKSTSGGTAAL.G 148 (Pep/81/-0.0551)<br>140 K.STSGGTAALGCLVK.D 153 (Try/67/0.1415)<br>142 T.SGGTAALGCLVK.D 153 (Try/52/0.0668)

		149 L.GCLVKDYFPEPVTVSW.N 164 (Chy/95/-0.0597)<br>149 L.GCLVKDYFPEPVTVSWNSGAL.T 169 (Chy/52/-0.1971)<br>149 L.GCLVKDYFPEPVTVSWNSGALTSGVH.T 174 (Chy/65/-0.158)<br>151 C.LVKDYFPEPVTVS.W 163 (Pep/59/0.1083)<br>152 L.VKDYFPEPVT.V 161 (Pep/33/0.0624)<br>154 K.DYFPEPVTVS.W 163 (ProK/56/-0.0063)<br>154 K.DYFPEPVTVSWN.S 165 (Try/63/-0.0503)<br>154 K.DYFPEPVTVSWNSGAL.T 169 (Try/77/0.0598)<br>154 K.DYFPEPVTVSWN*SGALTSGVHTFPAVLQ.S Deamidation(N) 181 (Try/61/0.0427)<br>162 T.VSWNSGALTSGVHTF.P 176 (Pep/59/0.1051)<br>163 V.SWNSGALTSGVHTF.P 176 (Pep/61/0.0795)<br>166 N.SGALTSGVHTFPAVLQSSGLY.S 186 (Chy/44/-0.032)<br>166 N.SGALTSGVHTFPAVLQ.S 181 (Try/29/0.0649)<br>166 N.SGALTSGVHTFPAVLQSS.G 183 (Try/58/0.0458)<br>166 N.SGALTSGVHTFPAVLQSSGLY.S 186 (Try/52/-0.044)<br>170 L.TSGVHTFPAVLQSSGLY.S 186 (Chy/93/0.0826)<br>171 T.SGVHTFPAVLQSSGLY.S 186 (Chy/63/0.1327)<br>175 H.TFPAVLQSSGLY.S 186 (Chy/64/-0.0163)<br>177 F.PAVLQ.S 181 (ProK/27/0.0383)<br>187 Y.SLSSVVTVPSSSLGTQTY.I 204 (Chy/41/-0.0127)<br>189 L.SSVVTVPSSSL.G 199 (ProK/39/0.0134)<br>189 L.SSVVTVPSSSLGTQTYICNVNHKPSNTK.V 216 (Try/46/-0.1276)<br>191 S.VVTVPSSSLGTQT.Y 203 (Pep/31/0.0194)<br>195 V.PSSSLGTQTY.I 204 (Chy/39/-0.0363)<br>197 S.SSLGTQTYICNVNHKPSNTK.V 216 (Try/54/-0.103)<br>205 Y.ICNVNHKPSNTK.V 216 (Try/39/0.0833)<br>216 T.KVDKKVEPK.S 224 (ProK/28/0.1511)<br>216 T.KVDKKVEPKSCDKTHTCPPCPAPEL.L 240 (Pep/51/-0.0807)<br>225 K.SCDKTHTCPPCPAPEL.L 240 (Pep/40/-0.1417)<br>225 K.SCDKTHTCPPCPAPELL.G 241 (Pep/42/-0.1744)<br>225 K.SCDKTHTCPPCPAPELLGGPS.V 245 (Pep/60/-0.1585)<br>229 K.THTCPPCPAPELLGGPSVF.L 247 (Try/52/-0.1036)<br>231 H.TCPPCPAPELLGGPSVF.L 247 (Chy/35/0.0504)<br>236 C.PAPELLGGPSVFLFPPKPK.D 254 (Try/63/-0.1237)<br>238 A.PELLGGPSVFLFPPK.P 252 (Try/56/0.1645)<br>238 A.PELLGGPSVFLFPPKPK.D 254 (Try/80/0.0116)<br>240 E.LLGGPSVFLFPPKPK.D 254 (Try/64/-0.0551)<br>241 L.LGGPSVFLFPPKPK.D 254 (Try/41/0.0216)<br>250 F.PPKPKDTLM.I 258 (Pep/37/0.0067)<br>259 M.ISRTPEVTC.V 267 (Pep/33/-0.0021)<br>262 R.TPEVTCVVVDVSHEDPEVK.F 280 (Try/85/-0.1463)<br>266 V.TCVVVDVSHEDPEVK.F 280 (Try/90/0.0559)<br>267 T.CVVVDVSHEDPEVK.F 280 (Try/49/-0.0474)<br>267 T.CVVVDVSHEDPEVK.F 280 (Pep/85/0.0492)<br>267 T.CVVVDVSHEDPEVKFN.W 282 (Pep/52/0.0159)<br>268 C.VVVDVSHEDPEVKF.N 281 (Pep/36/0.0336)<br>268 C.VVVDVSHEDPEVKFNW.Y 283 (Pep/68/-0.037)<br>270 V.VDVSHEDPEVKFNW.Y 283 (Pep/36/0.0482)

		271 V.DVSHEDPEVKFNW.Y 283 (Pep/46/0.1857)<br>272 D.VSHEDPEVKFNW.Y 283 (Pep/52/0.0838)<br>281 K.FNWYVDGVEVHNAK.T 294 (Try/79/0.0193)<br>282 F.NWYVDGVEVH.N 291 (Chy/34/-0.0635)<br>283 N.WYVDGV.E 288 (Pep/37/0.1192)<br>283 N.WYVDGVEVH.N 291 (Pep/40/0.0177)<br>284 W.YVDGVEVHNAK.T 294 (Try/43/0.0646)<br>287 D.GVEVHNAK.T 294 (Try/31/-0.0422)<br>307 Y.RVVSVLTVLHQDWLNGKEY.K 325 (Chy/53/-0.158)<br>307 Y.RVVSVLTVLHQ*DWLNGKEY.K Deamidation(Q) 325 (Chy/48/0.1911)<br>308 R.VVSVLTVLHQDWLNGK.E 323 (Chy/41/0.1726)<br>308 R.VVSVLTVLHQDWL.N 320 (Try/25/0.1658)<br>308 R.VVSVLTVLHQDWLN.G 321 (Try/26/0.0728)<br>308 R.VVSVLTVLHQDWLNGK.E 323 (Try/93/-0.0046)<br>308 R.VVSVLTVLHQDWLN*GK.E Deamidation(N) 323 (Try/107/0.106)<br>309 V.VSVLTVLHQDWLNGK.E 323 (Try/61/0.051)<br>309 V.VSVLTVLHQDWLN*GK.E Deamidation(N) 323 (Try/80/0.0094)<br>310 V.SVLTVLHQDWLNGK.E 323 (Chy/45/0.1481)<br>310 V.SVLTVLHQDWLNGK.E 323 (Try/88/0.1091)<br>310 V.SVLTVLHQDWLN*GK.E Deamidation(N) 323 (Try/80/0.048)<br>311 S.VLTVLHQDWLNGK.E 323 (Try/100/0.1773)<br>312 V.LTVLHQDWLNGK.E 323 (Try/34/0.0815)<br>313 L.TVLHQDWLN*GKEY.K Deamidation(N) 325 (Chy/43/0.1788)<br>313 L.TVLHQDWLNGK.E 323 (Try/49/0.119)<br>313 L.TVLHQDWLNGKE.Y 324 (Pep/61/0.069)<br>313 L.TVLHQDWLN*GKE.Y Deamidation(N) 324 (Pep/53/-0.0272)<br>314 T.VLHQDWLNGKEY.K 325 (Chy/40/0.1617)<br>315 V.LHQDWLNGKEY.K 325 (Chy/45/0.1616)<br>316 L.HQDWLNGKE.Y 324 (Pep/34/0.1073)<br>325 E.YKCKVSNKALPAPIE.K 339 (Pep/64/-0.0882)<br>325 E.YKCKVSNKALPAPIEKT.I 341 (Pep/48/-0.1174)<br>331 S.NKALPAPIEKTIS.K 343 (Pep/22/0.0924)<br>333 K.ALPAPIEKTISKAKGQPREPQVY.T 355 (Chy/37/0.0839)<br>333 K.ALPAPIEKTISK.A 344 (Pep/41/0.1793)<br>333 K.ALPAPIEKTISKAK.G 346 (Pep/31/0.1321)<br>337 A.PIEKTISKA.K 345 (Chy/48/-0.1023)<br>340 E.KTISKAKGQPREPQV.Y 354 (Pep/91/-0.0449)<br>341 K.TISKAKGQPREPQV.Y 354 (Pep/69/0.0068)<br>342 T.ISKAKGQPREPQVY.T 355 (Chy/32/0.0032)<br>342 T.ISKAKGQPREPQV.Y 354 (Pep/62/0.1569)<br>349 Q.PREPQVY.T 355 (Chy/31/-0.0012)<br>351 R.EPQVYTLPPSR.E 361 (Try/39/0.1076)<br>352 E.PQVYTLPPSR.E 361 (Try/50/0.0198)<br>366 T.KNQ*VSLTCLVKGFYPSDIAVEWESN*GQPENNYK.T Deamidation(NQ) 398 (Try/50/-0.0025)<br>367 K.NQVSLTCLVK.G 376 (Try/40/0.0019)<br>372 L.TCLVKGFYPSDIAVEW.E 387 (Chy/71/0.0529)<br>372 L.TCLVKGFYPSDIAVEWESNGQPENNY.K 397 (Chy/48/-0.0804)

		372 L.TCLVKGFYPSD.I 382 (Pep/35/0.1552)<br>373 T.CLVKGFYPSDIAVEW.E 387 (Chy/70/0.0144)<br>373 T.CLVKGFYPSDIAVEWESN*GQPENNY.K Deamidation(N) 397 (Chy/49/-0.0676)<br>377 K.GFYPSDIAVEWESNGQ*PENNYK.T Deamidation(Q) 398 (Chy/82/0.0324)<br>377 K.GFYPSDIAVEWESNGQPENNYK.T 398 (Try/75/-0.1596)<br>379 F.YPSDIAVE.W 386 (ProK/31/-0.0349)<br>380 Y.PSDIAVEWESNGQPENNYK.T 398 (Try/118/-0.1572)<br>385 A.VEWESN.G 390 (ProK/37/-0.0381)<br>387 E.WESNGQPENNYKTTPPVL.D 404 (Pep/39/-0.0871)<br>387 E.WESN*GQPENNYKTTPPVL.D Deamidation(N) 404 (Pep/51/-0.0429)<br>391 N.GQPENNYKTTPPVLDSDGSF.F 410 (Pep/46/-0.1065)<br>395 E.NNYKTTPPVLDSDGSF.F 410 (Pep/96/-0.0241)<br>397 N.YKTTPPVLDSDGSF.F 410 (Pep/94/-0.074)<br>398 Y.KTTPPVLDSDGSFF.L 411 (Chy/84/0.0015)<br>398 Y.KTTPPVLDSDGSF.F 410 (Pep/38/-0.0029)<br>399 K.TTPPVLDSDGSFFLYSK.L 415 (Chy/71/0.0073)<br>399 K.TTPPVLDSDGSF.F 410 (ProK/49/0.1227)<br>399 K.TTPPVLDSDGSFFLY.S 413 (Try/56/0.0249)<br>399 K.TTPPVLDSDGSFFLYSK.L 415 (Try/68/-0.1105)<br>401 T.PPVLDSDGSFFLYSK.L 415 (Chy/32/0.1658)<br>401 T.PPVLDSDGSFFLYSK.L 415 (Try/110/0.009)<br>402 P.PVLDSDGSFFLYSK.L 415 (Try/81/-0.0244)<br>414 Y.SKLTVDKSRWQQGNVF.S 429 (Chy/52/0.1544)<br>417 L.TVDKSRWQQGNVF.S 429 (Pep/33/0.0226)<br>428 N.VFSCSVM*HEALHNHYTQK.S Oxidation(M) 445 (Try/59/0.0367)<br>429 V.FSCSVMHEALHNHYTQK.S 445 (Try/43/0.1115)<br>430 F.SCSVMHEALHNHYTQK.S 445 (Try/54/-0.1437)<br>432 C.SVMHEALHNHY.T 442 (Chy/42/0.179)<br>432 C.SVMHEALHNHYTQK.S 445 (Try/61/-0.0604)<br>434 V.MHEALHNHYTQK.S 445 (Try/69/-0.0616)<br>435 M.HEALHNHYTQK.S 445 (Try/84/0.0645)<br>435 M.HEALHNHYTQKS.L 446 (Pep/46/0.1187)<br>435 M.HEALHNHYTQKSLSLSPG.K 452 (Pep/41/-0.0843)<br>439 L.HNHYTQKSLSLSPG.K 452 (Pep/47/-0.0516)
3	95%	1 -.EVQLVESGGGLVQPGGSLR.L 19 (Try/62/-0.0096)<br>1 -.EVQLVESGGGLVQPGGSL.R 18 (Chy/98/0.1397)<br>1 -.EVQLVESGGGLVQPGGSLRLSCAASGY.T 27 (Chy/90/-0.1479)<br>1 -.EVQLVESGGGLVQ*PGGSLRLSCAASGY.T Deamidation(Q) 27 (Chy/67/0.0922)<br>3 V.QLVESGGGLVQPGGSLR.L 19 (Try/112/0.015)<br>4 Q.LVESGGGLVQPGGSLRL.S 20 (Pep/88/0.1036)<br>5 L.VESGGGLVQPGGSLR.L 19 (Try/43/0.1025)<br>14 Q.PGGSLRLSCAASGY.T 27 (Chy/41/0.039)<br>14 Q.PGGSL.R 18 (Pep/31/0.0488)<br>19 L.RLSCAASGY.T 27 (Chy/42/-0.0058)<br>20 R.LSCAASGYTFTNYGMNWVR.Q 38 (Try/128/-0.1446)<br>20 R.LSCAASGYTFTNYGM*NWVR.Q Oxidation(M) 38 (Try/118/-0.1644)<br>21 L.SCAASGY.T 27 (Chy/35/0.009)<br>23 C.AASGYTFTNYGMNWVR.Q 38 (Try/87/-0.0029)

		28 Y.TFTNYGMNWVR.Q 38 (Try/57/0.1813)
		36 N.WVRQAPGKGLE.W 46 (Pep/36/0.1401)
		36 N.WVRQAPGKGLEW.V 47 (Pep/54/0.1163)
		36 N.WVRQAPGKGLEWVG.W 49 (Pep/48/0.0586)
		44 K.GLEWVGWINTY.T 54 (Try/69/-0.0187)
		44 K.GLEWVGWINTYTGEPTYAADFK.R 65 (Try/136/-0.1948)
		44 K.GLEWVGWINTYTGEPTYAADFKR.R 66 (Try/52/0.0908)
		44 K.GLEWVGWINTYTGEPTYAADFK.R 65 (Chy/150/-0.1842)
		48 W.VGWINTYTGEPTYAADFK.R 65 (Try/113/-0.1374)
		49 V.GWINTYTGEPTYAADFK.R 65 (Try/113/-0.0665)
		52 I.NTYTGEPTYAADFK.R 65 (Try/74/0.1411)
		55 Y.TGEPTYAADFK.R 65 (Try/73/0.1614)
		55 Y.TGEPTYAADF.K 64 (Chy/44/-0.0647)
		55 Y.TGEPTYAADFKRRFTF.S 70 (Chy/36/0.0059)
		58 E.PTYAADFK.R 65 (Try/43/0.0286)
		67 R.RFTFSLDTSK.S 76 (Try/51/0.0282)
		71 F.SLDTSKSTAY.L 80 (Chy/49/0.05)
		73 L.DTSKSTAY.L 80 (Chy/37/0.0065)
		77 K.STAYLQMN.S 84 (Try/29/-0.0153)
		77 K.STAYLQMNSLR.A 87 (Try/69/-0.0075)
		77 K.STAYLQM*NSLR.A Oxidation(M) 87 (Try/62/0.138)
		81 Y.LQMNSLRAEDTAVYY.C 95 (Chy/53/0.0071)
		87 L.RAEDTAVY.Y 94 (Chy/43/-0.0053)
		88 R.AEDTAVYYCAK.Y 98 (Try/69/0.0533)
		94 V.YYCAKYPHYYGSS.H 106 (Pep/34/0.0305)
		95 Y.YCAKYPHYYGSS.H 106 (Pep/37/0.0899)
		109 W.YFDVWGQGTL.V 118 (Chy/35/-0.0894)
		114 W.GQGTLVTVSSASTKGPSVFPL.A 134 (Chy/34/-0.0711)
		119 L.VTVSSASTKGPSVF.P 132 (Pep/75/0.1456)
		119 L.VTVSSASTKGPSVFPLAPSS.K 138 (Pep/32/-0.0295)
		121 T.VSSASTKGPSVFPLAPSSKSTSGGTAAL.G 148 (Chy/43/-0.0294)
		121 T.VSSASTKGPSVFPLAPSSKSTSGGTAAL.G 148 (Pep/48/-0.0675)
		125 A.STKGPSVFPLAPSSKSTSGGTAAL.G 148 (Pep/70/-0.0982)
		127 T.KGPSVFPLAPSSKSTSGGTAAL.G 148 (Chy/73/-0.0291)
		128 K.GPSVFPLAPSSK.S 139 (Try/59/-0.0321)
		133 F.PLAPSSKSTSGGTAAL.G 148 (Pep/63/0.0703)
		140 K.STSGGTAALGCLVK.D 153 (Try/87/0.0941)
		141 S.TSGGTAALGCLVK.D 153 (Try/89/0.1919)
		142 T.SGGTAALGCLVK.D 153 (Try/52/-0.0676)
		146 T.AALGCLVKDYFPEPVTVSW.N 164 (Chy/36/0.0644)
		149 L.GCLVKDYFPEPVTVSW.N 164 (Chy/89/-0.0555)
		149 L.GCLVKDYFPEPVTVSWNSGAL.T 169 (Chy/68/-0.0361)
		149 L.GCLVKDYFPEPVTVSWNSGALTSGVH.T 174 (Chy/65/-0.1676)
		149 L.GCLVKDYFPEPVT.V 161 (Pep/51/0.1526)
		150 G.CLVKDYFPEPVTVSW.N 164 (Chy/55/-0.0745)
		154 K.DYFPEPVTVSWN.S 165 (Try/58/-0.0819)
		154 K.DYFPEPVTVSWNSGAL.T 169 (Try/76/0.0338)
		157 F.PEPVTVSW.N 164 (Chy/42/-0.04)

		162 T.VSWNSGALTSGVHTF.P 176 (Pep/54/0.1091)<br>165 W.N*SGALTSGVHTFPAVLQSSGLY.S Deamidation(N) 186 (Chy/55/-0.113)<br>166 N.SGALTSGVHTFPAVLQS.S 182 (Try/78/-0.0236)<br>166 N.SGALTSGVHTFPAVLQSS.G 183 (Try/49/-0.073)<br>166 N.SGALTSGVHTFPAVLQSSGLY.S 186 (Try/82/-0.1222)<br>166 N.SGALTSGVHTFPAVLQSSGLYSL.S 188 (Try/85/-0.1999)<br>166 N.SGALTSGVHTFPAVLQ*SSGLYSLSSVVTVPS.S Deamidation(Q) 196 (Try/41/-0.0192)<br>170 L.TSGVHTFPAVLQSSGLY.S 186 (Chy/68/0.0678)<br>170 L.TSGVHTFPAVL.Q 180 (Pep/38/-0.0174)<br>171 T.SGVHTFPAVLQSSGLY.S 186 (Chy/70/0.1587)<br>175 H.TFPAVLQSSGLY.S 186 (Chy/56/0.0225)<br>191 S.VVTVPSSSL.G 199 (Pep/26/0.162)<br>195 V.PSSSLGTQTY.I 204 (Chy/37/-0.0448)<br>197 S.SSLGTQTYICNVNHKPSNTK.V 216 (Try/48/-0.0984)<br>203 Q.TYICNVNHKPSNTK.V 216 (Try/46/-0.0109)<br>205 Y.ICNVNHKPSNTK.V 216 (Try/34/0.0377)<br>229 K.THTCPPCPAPELLGGPSVF.L 247 (Try/42/-0.1168)<br>229 K.THTCPPCPAPELLGGPSVFLFPPKPK.D 254 (Try/44/-0.1876)<br>231 H.TCPPCPAPELLGGPSVF.L 247 (Chy/42/0.13)<br>238 A.PELLGGPSVFLFPPKPK.D 254 (Try/72/0.0074)<br>239 P.ELLGGPSVFLFPPKPK.D 254 (Try/39/0.1143)<br>240 E.LLGGPSVFLFPPKPK.D 254 (Try/67/-0.0001)<br>241 L.LGGPSVFLFPPKPK.D 254 (Try/38/-0.0032)<br>247 V.FLFPPKPKDTLM.I 258 (Pep/24/0.185)<br>248 F.LFPPKPKDTLM.I 258 (Pep/27/0.0962)<br>250 F.PPKPKDTLM.I 258 (Pep/36/0.1305)<br>255 K.DTLMISR.T 261 (Try/34/0.0045)<br>259 M.ISRTPEVTC.V 267 (Pep/50/0.1281)<br>262 R.TPEVTCVVVDVSHEDPEVK.F 280 (Try/92/-0.1743)<br>263 T.PEVTCVVVDVSHEDPEVK.F 280 (Try/103/-0.1929)<br>264 P.EVTCVVVDVSHEDPEVK.F 280 (Try/82/-0.1505)<br>266 V.TCVVVDVSHEDPEVK.F 280 (Try/96/-0.1105)<br>268 C.VVVDVSHEDPEVKF.N 281 (Pep/51/0.038)<br>268 C.VVVDVSHEDPEVKFNW.Y 283 (Pep/53/-0.0536)<br>269 V.VVDVSHEDPEVKFNW.Y 283 (Pep/51/0.1162)<br>272 D.VSHEDPEVKFNW.Y 283 (Pep/76/0.0422)<br>281 K.FNWYVDGVEVH.N 291 (Try/49/0.1653)<br>281 K.FNWYVDGVEVHNAK.T 294 (Try/100/0.1483)<br>282 F.NWYVDGVEVHNAK.T 294 (Try/82/-0.005)<br>282 F.NWYVDGVEVH.N 291 (Chy/34/0.1837)<br>284 W.YVDGVEVHNAK.T 294 (Try/53/0.0328)<br>307 Y.RVVSVLTVLHQDW.L 319 (Chy/71/-0.0385)<br>307 Y.RVVSVLTVLHQDWLNGKEY.K 325 (Chy/63/-0.1678)<br>307 Y.RVVSVLTVLHQ*DWLNGKEY.K Deamidation(Q) 325 (Chy/51/0.1698)<br>308 R.VVSVLTVLHQDWLN.G 321 (Try/28/0.0896)<br>308 R.VVSVLTVLHQDWLNGK.E 323 (Try/93/-0.0438)<br>308 R.VVSVLTVLHQDWLN*GK.E Deamidation(N) 323 (Try/97/-0.0126)<br>309 V.VSVLTVLHQDWLNGK.E 323 (Try/68/0.0384)

		309 V.VSVLTVLHQDWLN*GK.E Deamidation(N) 323 (Try/71/0.0182)<br>310 V.SVLTVLHQDWLNGK.E 323 (Try/104/0.0523)<br>310 V.SVLTVLHQDWLN*GK.E Deamidation(N) 323 (Try/79/0.0502)<br>311 S.VLTVLHQDWLNGK.E 323 (Try/87/0.1035)<br>311 S.VLTVLHQDWLN*GK.E Deamidation(N) 323 (Try/88/-0.0109)<br>312 V.LTVLHQDWLNGK.E 323 (Try/74/0.1825)<br>312 V.LTVLHQDWLN*GK.E Deamidation(N) 323 (Try/86/0.0467)<br>313 L.TVLHQDWLN*GK.E Deamidation(N) 323 (Try/47/0.0364)<br>313 L.TVLHQDWLNGKEY.K 325 (Chy/79/0.0144)<br>313 L.TVLHQDWLN*GKEY.K Deamidation(N) 325 (Chy/47/0.1744)<br>314 T.VLHQDWLNGKEY.K 325 (Chy/40/0.1489)<br>315 V.LHQDWLNGKEY.K 325 (Chy/54/0.0554)<br>325 E.YKCKVSNKALPAPIE.K 339 (Pep/93/-0.0124)<br>338 P.IEKTISKAKGQPREPQV.Y 354 (Pep/56/-0.1994)<br>340 E.KTISKAKGQPREPQV.Y 354 (Pep/58/-0.1253)<br>344 S.KAKGQPREPQVY.T 355 (Chy/36/0.1083)<br>351 R.EPQVYTLPPSR.E 361 (Try/44/0.0214)<br>352 E.PQVYTLPPSR.E 361 (Try/53/0.081)<br>366 T.KNQ*VSLTCLVKGFYPSDIAVEWESN*GQPENNYK.T 2 Deamidation(NQ) 398 (Try/52/-0.064[illegible]<br>367 K.NQVSLTCLVK.G 376 (Try/44/0.0199)<br>372 L.TCLVKGFYPSDIAVEW.E 387 (Chy/59/0.0845)<br>372 L.TCLVKGFYPSDIAVEWESN*GQPENNY.K Deamidation(N) 397 (Chy/56/-0.1137)<br>373 T.CLVKGFYPSDIAVEW.E 387 (Chy/65/0.03)<br>373 T.CLVKGFYPSDIAVEWESNGQPENNY.K 397 (Chy/64/-0.0304)<br>375 L.VKGFYPSDIAVEWESNGQPENNY.K 397 (Chy/37/0.0732)<br>377 K.GFYPSDIAVEWESNGQPENNYK.T 398 (Try/86/-0.1938)<br>377 K.GFYPSDIAVEWESNGQ*PENNYK.T Deamidation(Q) 398 (Try/50/0.1353)<br>380 Y.PSDIAVEWESNGQPENNYK.T 398 (Try/111/-0.0606)<br>387 E.WESNGQPENNYKTTPPVL.D 404 (Pep/44/-0.1409)<br>397 N.YKTTPPVL.D 404 (Pep/29/0.0063)<br>398 Y.KTTPPVLDSDGSFFLY.S 413 (Chy/64/0.1625)<br>399 K.TTPPVLDSDGSFFLY.S 413 (Try/62/0.0751)<br>399 K.TTPPVLDSDGSFFLYSK.L 415 (Try/88/-0.1527)<br>400 T.TPPVLDSDGSFFLYSK.L 415 (Try/118/-0.0392)<br>401 T.PPVLDSDGSFFLYSK.L 415 (Try/123/0.0534)<br>402 P.PVLDSDGSFFLYSK.L 415 (Try/68/0.0058)<br>412 F.LYSKL.T 416 (Chy/17/0.1468)<br>414 Y.SKLTVDKSRWQQGNVF.S 429 (Chy/60/-0.0482)<br>417 L.TVDKSRWQQGNVF.S 429 (Pep/33/0.0938)<br>423 R.WQQGNVFSCSVMHEALHN*HYTQK.S Deamidation(N) 445 (Try/64/0.164)<br>423 R.WQQGNVFSCSVM*HEALHNHYTQK.S Oxidation(M) 445 (Try/44/-0.1766)<br>428 N.VFSCSVMHEALHNHYTQK.S 445 (Try/53/0.1226)<br>429 V.FSCSVMHEALHNHYTQK.S 445 (Try/39/0.1199)<br>430 F.SCSVMHEALHNHYTQK.S 445 (Try/59/-0.1117)<br>430 F.SCSVMHEALHNHY.T 442 (Chy/54/0.0631)<br>430 F.SCSVMHEALHNHYTQKSLS.L 448 (Pep/36/-0.1638)<br>431 S.CSVMHEALHNHYTQK.S 445 (Try/34/0.011)<br>432 C.SVMHEALHNHYTQK.S 445 (Try/71/0.0082)

		432 C.SVMHEALHNHY.T 442 (Chy/32/0.1812)<br>434 V.MHEALHNHYTQK.S 445 (Try/80/0.0058)<br>435 M.HEALHNHYTQK.S 445 (Try/85/0.0741)<br>435 M.HEALHNHYTQKSLSLSPG.K 452 (Pep/41/-0.1197)
4	83%	20 R.LSCAASGYTFTNYGMNWVR.Q 38 (Try/124/-0.1662)<br>20 R.LSCAASGYTFTNYGM*NWVR.Q Oxidation(M) 38 (Try/102/-0.1352)<br>23 C.AASGYTFTNYGMNWVR.Q 38 (Try/89/-0.0181)<br>28 Y.TFTNYGMNWVR.Q 38 (Try/41/0.1049)<br>28 Y.TFTNYGM*NWVR.Q Oxidation(M) 38 (Try/38/0.0296)<br>44 K.GLEWVGWINTYTGEPTYAADFK.R 65 (Try/98/-0.1764)<br>48 W.VGWINTYTGEPTYAADFK.R 65 (Try/77/0.0264)<br>49 V.GWINTYTGEPTYAADFK.R 65 (Try/114/0.0147)<br>51 W.INTYTGEPTYAADFK.R 65 (Try/98/0.0346)<br>51 W.IN*TYTGEPTYAADFK.R Deamidation(N) 65 (Try/56/0.0174)<br>52 I.NTYTGEPTYAADFK.R 65 (Try/64/0.0995)<br>53 N.TYTGEPTYAADFK.R 65 (Try/75/0.0894)<br>55 Y.TGEPTYAADFK.R 65 (Try/35/0.0404)<br>58 E.PTYAADFK.R 65 (Try/31/0.0194)<br>67 R.RFTFSLDTSK.S 76 (Try/49/0.1258)<br>77 K.STAYLQMNSLR.A 87 (Try/62/0.1127)<br>77 K.STAYLQM*NSLR.A Oxidation(M) 87 (Try/62/0.1134)<br>88 R.AEDTAVYYCAK.Y 98 (Try/70/0.1203)<br>128 K.GPSVFPLAPSSK.S 139 (Try/39/0.1917)<br>140 K.STSGGTAALGCLVK.D 153 (Try/81/0.0469)<br>141 S.TSGGTAALGCLVK.D 153 (Try/61/0.0887)<br>142 T.SGGTAALGCLVK.D 153 (Try/55/-0.0694)<br>154 K.DYFPEPVTVSWNSGAL.T 169 (Try/68/0.1052)<br>166 N.SGALTSGVHTFPAVLQ*SSGLYSLSSVVTVPSSSLGTQTY.I Deamidation(Q) 204 (Try/45/-0.00[illegible]<br>195 V.PSSSLGTQTYICNVNHK.P 211 (Try/39/-0.1594)<br>195 V.PSSSLGTQTYICNVNHKPSNTK.V 216 (Try/37/0.1593)<br>229 K.THTCPPCPAPELLGGPSVF.L 247 (Try/63/-0.0846)<br>236 C.PAPELLGGPSVFLFPPKPK.D 254 (Try/80/-0.1109)<br>238 A.PELLGGPSVFLFPPKPK.D 254 (Try/77/0.0444)<br>262 R.TPEVTCVVVDVSHEDPEVK.F 280 (Try/80/-0.1967)<br>263 T.PEVTCVVVDVSHEDPEVK.F 280 (Try/72/0.0171)<br>265 E.VTCVVVDVSHEDPEVK.F 280 (Try/99/-0.1617)<br>266 V.TCVVVDVSHEDPEVK.F 280 (Try/66/-0.1179)<br>267 T.CVVVDVSHEDPEVK.F 280 (Try/84/-0.1344)<br>281 K.FNWYVDGVEVHNAK.T 294 (Try/78/0.0063)<br>282 F.NWYVDGVEVHNAK.T 294 (Try/64/0.039)<br>308 R.VVSVLTVLHQDWLNGK.E 323 (Try/93/0.0166)<br>308 R.VVSVLTVLHQDWLN*GK.E Deamidation(N) 323 (Try/91/0.0072)<br>309 V.VSVLTVLHQDWLNGK.E 323 (Try/71/0.0766)<br>309 V.VSVLTVLHQDWLN*GK.E Deamidation(N) 323 (Try/70/0.071)<br>310 V.SVLTVLHQDWLNGK.E 323 (Try/94/0.0847)<br>310 V.SVLTVLHQDWLN*GK.E Deamidation(N) 323 (Try/73/0.0874)<br>311 S.VLTVLHQDWLN*GK.E Deamidation(N) 323 (Try/81/0.1077)<br>313 L.TVLHQDWLN*GK.E Deamidation(N) 323 (Try/61/0.078)

		351 R.EPQVYTLPPSR.E 361 (Try/50/0.0754)<br>352 E.PQVYTLPPSR.E 361 (Try/56/0.0752)<br>367 K.NQVSLTCLVK.G 376 (Try/41/0.1271)<br>377 K.GFYPSDIAVEWESNGQPENNYK.T 398 (Try/87/-0.1406)<br>380 Y.PSDIAVEWESN*GQPENNYK.T Deamidation(N) 398 (Try/42/-0.1548)<br>399 K.TTPPVLDSDGSFFLYSK.L 415 (Try/74/0.0839)<br>401 T.PPVLDSDGSFFLYSK.L 415 (Try/106/0.0226)<br>423 R.WQQGNVFSCSVM*HEALHNHYTQK.S Oxidation(M) 445 (Try/61/-0.1154)<br>428 N.VFSCSVMHEALHNHYTQK.S 445 (Try/45/0.1628)<br>430 F.SCSVMHEALHNHYTQK.S 445 (Try/43/-0.1353)<br>432 C.SVMHEALHNHYTQK.S 445 (Try/60/0.0138)<br>434 V.MHEALHNHYTQK.S 445 (Try/45/-0.0584)<br>435 M.HEALHNHYTQK.S 445 (Try/47/0.0269)
5	100%	1 -.DIQMTQSPSSLSASVGDR.V 18 (Try/147/-0.1515)<br>1 -.DIQMTQSPSSLSASVGDR.V 18 (Try/69/-0.104)<br>1 -.DIQMTQSPSSLSASVGDRVTITCSASQDISNY.L 32 (Chy/47/-0.1008)<br>1 -.DIQM*TQSPSSL.S Oxidation(M) 11 (Pep/33/-0.0369)<br>2 D.IQMTQSPSSLSASVGDR.V 18 (Try/95/0.0565)<br>3 I.QMTQSPSSLSASVGDR.V 18 (Try/80/-0.0013)<br>4 Q.MTQSPSSLSASVGDR.V 18 (Try/63/-0.1073)<br>5 M.TQSPSSLSASVGDRVTIT.C 22 (Pep/43/-0.102)<br>6 T.QSPSSLSASVGDRVT.I 20 (ProK/38/-0.0624)<br>12 L.SASVGDRVTITCSASQDISNYLNW.Y 35 (Chy/64/0.0862)<br>12 L.SASVGDRVTIT.C 22 (ProK/30/-0.0379)<br>12 L.SASVGDRVTIT.C 22 (Pep/31/-0.0379)<br>12 L.SASVGDRVTITCSASQ.D 27 (Pep/67/-0.0905)<br>12 L.SASVGDRVTITCSASQD.I 28 (Pep/53/-0.1008)<br>12 L.SASVGDRVTITCSASQDISN.Y 31 (Pep/90/-0.1428)<br>12 L.SASVGDRVTITCSASQDISNY.L 32 (Pep/65/-0.0971)<br>14 A.SVGDRVTITCSASQDISN.Y 31 (Pep/71/-0.1137)<br>19 R.VTITCSASQDISNY.L 32 (Try/74/0.1292)<br>19 R.VTITCSASQDISNYLNWY.Q 36 (Try/114/-0.1615)<br>19 R.VTITCSASQDISNYLNWYQQKPGK.A 42 (Try/77/-0.0286)<br>19 R.VTITCSASQDISN*YLNWYQQKPGK.A Deamidation(N) 42 (Try/137/0.1189)<br>20 V.TITCSASQDISNYLNWYQQKPGK.A 42 (Try/57/-0.0628)<br>21 T.ITCSASQDISNYLNWYQQKPGK.A 42 (Try/66/-0.1469)<br>24 C.SASQDISNYLNWYQQK.P 39 (Try/74/-0.0707)<br>24 C.SASQDISNYLNWYQQKPGK.A 42 (Try/85/-0.1355)<br>24 C.SASQDISNY.L 32 (Chy/33/-0.0489)<br>26 A.SQDISNY.L 32 (ProK/36/-0.0669)<br>33 Y.LNWYQQKPGKAPK.V 45 (ProK/30/0.0135)<br>33 Y.LNWYQQKPGKAPK.V 45 (Pep/36/0.0349)<br>35 N.WYQQKPGKAPK.V 45 (ProK/46/0.1213)<br>36 W.YQQKPGKAPKVL.I 47 (Chy/26/0.0758)<br>36 W.YQQKPGKAPKV.L 46 (ProK/28/0.1344)<br>36 W.YQQKPGKAPKVL.I 47 (Pep/39/-0.0118)<br>36 W.YQQKPGKAPKVLI.Y 48 (Pep/28/0.0187)<br>36 W.YQQKPGKAPKVLIY.F 49 (Pep/43/0.0572)

		46 K.VLIYFTSSLHSGVPSR.F 61 (Try/85/-0.0187)<br>47 V.LIYFTSSLHSGVPSR.F 61 (Try/100/-0.0775)<br>48 L.IYFTSSLHSGVPSR.F 61 (Try/114/0.0524)<br>48 L.IYFTSSLHSGVPSRF.S 62 (Pep/48/-0.0921)<br>49 I.YFTSSLHSGVPSR.F 61 (Try/94/0.023)<br>50 Y.FTSSLHSGVPSR.F 61 (Try/84/0.1433)<br>51 F.TSSLHSGVPSR.F 61 (Try/63/0.0604)<br>51 F.TSSLHSGVPSRF.S 62 (Chy/42/0.1207)<br>55 L.HSGVPSR.F 61 (ProK/28/0.0934)<br>55 L.HSGVPSRFSGSGSGTD.F 70 (Pep/75/0.0508)<br>55 L.HSGVPSRFSGSGSGTDF.T 71 (Pep/69/-0.0834)<br>72 F.TLTISSLQPEDFATY.Y 86 (Chy/62/0.0771)<br>74 L.TISSLQPEDFATYY.C 87 (Chy/68/0.1713)<br>77 S.SLQPEDFAT.Y 85 (ProK/37/-0.0297)<br>80 Q.PEDFAT.Y 85 (ProK/42/-0.097)<br>87 Y.YCQQYSTVPWTFGQGTK.V 103 (Pep/55/-0.117)<br>88 Y.CQQYSTVPWTFGQGTK.V 103 (Try/62/-0.1047)<br>91 Q.YSTVPWTFGQGTKVE.I 105 (Pep/41/0.0863)<br>91 Q.YSTVPWTFGQGTKVEIKRTVAA.P 112 (Pep/54/-0.12)<br>92 Y.STVPWTFGQGTK.V 103 (Try/45/0.008)<br>94 T.VPWTFGQGTK.V 103 (Try/38/0.1685)<br>95 V.PWTFGQGTK.V 103 (Try/36/0.0199)<br>97 W.TFGQGTKVEIKRT.V 109 (Chy/31/0.1204)<br>97 W.TFGQGTKVEIKRTVAAPSVF.I 116 (Chy/57/-0.0983)<br>99 F.GQGTKVEIKRTVAAPSVF.I 116 (Chy/68/-0.143)<br>99 F.GQGTKVEIKRTVAA.P 112 (Pep/53/-0.0565)<br>103 T.KVEIKRTVAAPSVF.I 116 (Chy/44/0.1602)<br>108 K.RTVAAPSVFIFPPSDEQLK.S 126 (Try/68/-0.1517)<br>109 R.TVAAPSVFIFPPSDEQLK.S 126 (Try/79/0.1036)<br>111 V.AAPSVFIFPPSDEQLK.S 126 (Try/69/-0.0865)<br>112 A.APSVFIFPPSDEQLK.S 126 (Try/60/-0.1932)<br>113 A.PSVFIFPPSDEQLK.S 126 (Try/85/0.0521)<br>115 S.VFIFPPSDEQLKSG.T 128 (ProK/47/0.0374)<br>115 S.VFIFPPSDEQLK.S 126 (Pep/43/0.0741)<br>115 S.VFIFPPSDEQLKSG.T 128 (Pep/48/-0.0088)<br>116 V.FIFPPSD.E 122 (Pep/29/0.0299)<br>117 F.IFPPSDEQLKSGTASVVCL.L 135 (Chy/37/0.0454)<br>117 F.IFPPSDEQLKSGTASVVCLL.N 136 (Chy/63/-0.0764)<br>118 I.FPPSDEQLK.S 126 (Try/35/-0.0717)<br>119 F.PPSDEQLK.S 126 (Try/55/0.0024)<br>119 F.PPSDEQLKSGTASVVCLL.N 136 (Chy/93/0.1479)<br>119 F.PPSDEQLK.S 126 (ProK/51/0.052)<br>119 F.PPSDEQLKS.G 127 (ProK/38/0.038)<br>119 F.PPSDEQLKSG.T 128 (ProK/42/0.1317)<br>119 F.PPSDEQLKSGT.A 129 (ProK/29/0.1394)<br>119 F.PPSDEQLKSGTA.S 130 (ProK/48/0.1607)<br>120 P.PSDEQLKS.G 127 (ProK/47/0.0105)<br>120 P.PSDEQLKSG.T 128 (ProK/52/-0.006)

		126 L.KSGTASVVCLLNNFYPREAKVQ*W.K Deamidation(Q) 148 (Chy/48/-0.0673)<br>127 K.SGTASVVCLLNNFYPR.E 142 (Try/59/-0.0103)<br>127 K.SGTASVVCLLN*NFYPR.E Deamidation(N) 142 (Try/65/0.0651)<br>130 T.ASVVCLLNNFYPR.E 142 (Try/40/-0.1147)<br>133 V.VCLLNNFYPRE.A 143 (Pep/35/0.1084)<br>133 V.VCLLN*NFYPRE.A Deamidation(N) 143 (Pep/36/0.0156)<br>134 V.CLLNNFYPREAKVQW.K 148 (Chy/27/0.198)<br>137 L.NNFYPREAKVQW.K 148 (Chy/65/-0.0407)<br>140 F.YPREAKVQWKVDNALQSGNSQE.S 161 (Pep/65/-0.1336)<br>144 E.AKVQWKVDNALQSGNSQE.S 161 (Pep/78/-0.1415)<br>148 Q.WKVDNA.L 153 (ProK/28/-0.0778)<br>149 W.KVDNALQSGNSQE.S 161 (Pep/92/0.1469)<br>150 K.VDNALQSGNSQESVTEQDSK.D 169 (Try/135/-0.134)<br>150 K.VDN*ALQSGNSQESVTEQDSK.D Deamidation(N) 169 (Try/76/-0.1906)<br>153 N.ALQSGNSQESVTEQDSK.D 169 (Try/37/-0.1403)<br>159 N.SQESVTEQDSKDST.Y 172 (ProK/34/-0.0624)<br>161 Q.ESVTEQDSKDSTY.S 173 (ProK/68/-0.0855)<br>162 E.SVTEQDSKDST.Y 172 (Pep/35/0.1966)<br>162 E.SVTEQDSKDSTY.S 173 (Pep/32/0.1839)<br>162 E.SVTEQDSKDSTYSL.S 175 (Pep/46/-0.0434)<br>162 E.SVTEQDSKDSTYSLSST.L 178 (Pep/42/-0.053)<br>162 E.SVTEQDSKDSTYSLSSTL.T 179 (Pep/79/-0.1178)<br>170 K.DSTYSLSSTLTLSK.A 183 (Try/96/0.0193)<br>179 T.LTLSKADYEKHKVY.A 192 (Pep/32/0.0332)<br>179 T.LTLSKADYEKHKVYACE.V 195 (Pep/32/0.0795)<br>180 L.TLSKADYEKHKVY.A 192 (Chy/58/-0.1384)<br>180 L.TLSKADYEKHKVY.A 192 (Pep/40/-0.1048)<br>180 L.TLSKADYEKHKVYACE.V 195 (Pep/88/0.0043)<br>182 L.SKADYEKHKVY.A 192 (Chy/38/0.0716)<br>182 L.SKADYEKHKVY.A 192 (Pep/41/0.129)<br>182 L.SKADYEKHKVYACE.V 195 (Pep/84/-0.0276)<br>191 K.VYACEVTHQGLSSPVTK.S 207 (Try/84/0.0882)<br>192 V.YACEVTHQGLSSPVTK.S 207 (Try/104/0.0096)<br>193 Y.ACEVTHQGLSSPVTK.S 207 (Try/106/0.0479)<br>193 Y.ACEVTHQGL.S 201 (Chy/42/-0.0224)<br>193 Y.ACEVTHQGLSSPVTK.S 207 (Chy/89/0.0715)<br>193 Y.ACEVTHQGLSSPVTKSF.N 209 (Chy/36/-0.0545)<br>193 Y.ACEVTHQGLSSPVTKSFNRGEC.- 214 (Pep/41/-0.052)<br>194 A.CEVTHQGLSSPVTK.S 207 (Try/68/0.0116)<br>194 A.CEVTHQGL.S 201 (Chy/36/-0.0027)<br>194 A.CEVTHQGLSSPVTK.S 207 (Chy/53/0.1138)<br>195 C.EVTHQGLSSPVTK.S 207 (Try/57/0.0353)<br>196 E.VTHQGLSSPVTKS.F 208 (Pep/32/0.1453)<br>196 E.VTHQGLSSPVTKSFNRGEC.- 214 (Pep/57/-0.0923)<br>202 L.SSPVTKSFNRGEC.- 214 (Chy/62/0.029)<br>204 S.PVTKSFNRGEC.- 214 (Chy/39/0.0797)<br>204 S.PVTKSFNRGEC.- 214 (Pep/39/0.0877)
6	88%	1 -.DIQMTQSPSSLSASVGDR.V 18 (Try/142/-0.1007)

		1 -.DIQM*TQSPSSLSASVGDR.V Oxidation(M) 18 (Try/111/-0.1384)<br>1 -.DIQMTQSPSSLSASVGDRVTITCSASQDISNY.L 32 (Chy/71/-0.1875)<br>3 I.QMTQSPSSLSASVGDR.V 18 (Try/103/0.1589)<br>3 I.QM*TQSPSSLSASVGDR.V Oxidation(M) 18 (Try/86/0.011)<br>4 Q.MTQSPSSLSASVGDR.V 18 (Try/79/0.0483)<br>5 M.TQSPSSLSASVGDR.V 18 (Try/32/0.0604)<br>8 S.PSSLSASVGDR.V 18 (Try/80/-0.0951)<br>12 L.SASVGDRVTITC.S 23 (Chy/25/0.1827)<br>12 L.SASVGDRVTITCSASQDISNY.L 32 (Chy/110/-0.1119)<br>12 L.SASVGDRVTITCSASQD.I 28 (Pep/54/0.1336)<br>12 L.SASVGDRVTITCSASQDISN.Y 31 (Pep/98/-0.0464)<br>12 L.SASVGDRVTITCSASQDISNY.L 32 (Pep/86/-0.0339)<br>19 R.VTITCSASQDISNYLNWY.Q 36 (Try/104/-0.1217)<br>19 R.VTITCSASQDISNYLNWYQQK.P 39 (Try/103/-0.1596)<br>19 R.VTITCSASQDISNYLNWYQQKPGK.A 42 (Try/99/-0.1276)<br>19 R.VTITCSASQDISNYLN*WYQQKPGK.A Deamidation(N) 42 (Try/107/0.0619)<br>20 V.TITCSASQDISNYLNWYQQKPGK.A 42 (Try/77/-0.142)<br>21 T.ITCSASQDISNYLNWYQQKPGK.A 42 (Try/77/0.004)<br>22 I.TCSASQDISNYLNWYQQKPGK.A 42 (Try/46/0.1779)<br>24 C.SASQDISNYLNWYQQKPGK.A 42 (Try/56/-0.1305)<br>24 C.SASQDISNYLNWYQQKPGK.A 42 (Try/90/-0.1305)<br>24 C.SASQDISNY.L 32 (Chy/30/-0.0786)<br>28 Q.DISNYLNWYQQKPGK.A 42 (Try/37/-0.0143)<br>28 Q.DISNYLNWYQQKPGK.A 42 (Try/55/-0.0143)<br>30 I.SNYLNWYQQKPGK.A 42 (Try/59/0.0881)<br>32 N.YLNWYQQKPGKAPKVL.I 47 (Pep/58/0.0971)<br>33 Y.LNWYQQKPGKAPKVL.I 47 (Pep/58/0.1201)<br>33 Y.LNWYQQKPGKAPKVLIY.F 49 (Pep/31/0.1113)<br>36 W.YQQKPGKAPKVL.I 47 (Chy/17/0.1708)<br>36 W.YQQKPGKAPKVL.I 47 (Pep/32/0.1142)<br>36 W.YQQKPGKAPKVLIY.F 49 (Pep/40/0.1656)<br>46 K.VLIYFTSSLHSGVPSR.F 61 (Try/102/-0.0613)<br>47 V.LIYFTSSLHSGVPSR.F 61 (Try/97/0.0155)<br>48 L.IYFTSSLHSGVPSR.F 61 (Try/109/-0.0134)<br>48 L.IYFTSSLHSGVPSRF.S 62 (Chy/86/-0.0157)<br>48 L.IYFTSSL.H 54 (Pep/32/0.0367)<br>49 I.YFTSSLHSGVPSR.F 61 (Try/87/0.0494)<br>49 I.YFTSSLHSGVPSRF.S 62 (Chy/54/0.0264)<br>50 Y.FTSSLHSGVPSRF.S 62 (Chy/74/0.1183)<br>51 F.TSSLHSGVPSR.F 61 (Try/79/-0.0228)<br>51 F.TSSLHSGVPSRF.S 62 (Chy/49/0.0451)<br>52 T.SSLHSGVPSR.F 61 (Try/37/-0.0368)<br>53 S.SLHSGVPSR.F 61 (Try/40/-0.0757)<br>55 L.HSGVPSR.F 61 (Try/27/0.0092)<br>55 L.HSGVPSRFSGSGSGTD.F 70 (Pep/90/-0.0124)<br>55 L.HSGVPSRFSGSGSGTDF.T 71 (Pep/76/0.1618)<br>56 H.SGVPSRF.S 62 (Chy/27/-0.0295)<br>87 Y.YCQQYSTVPWTFGQGTKV.E 104 (Pep/50/0.0433)

		87 Y.YCQQYSTVPWTFGQGTKVE.I 105 (Pep/81/-0.1527)<br>88 Y.CQQYSTVPWTFGQGTK.V 103 (Try/82/-0.1737)<br>91 Q.YSTVPWTFGQGTKV.E 104 (Pep/35/0.0641)<br>91 Q.YSTVPWTFGQGTKVE.I 105 (Pep/38/0.1031)<br>91 Q.YSTVPWTFGQGTKVEIKRTVAA.P 112 (Pep/54/-0.131)<br>92 Y.STVPWTFGQGTK.V 103 (Try/41/0.0292)<br>92 Y.STVPWTFGQ*GTKVEIKRTVAAPSVF.I Deamidation(Q) 116 (Chy/27/0.1082)<br>92 Y.STVPWTFGQGTKVEIKRTVAA.P 112 (Pep/47/-0.0252)<br>94 T.VPWTFGQGTK.V 103 (Try/30/0.0485)<br>95 V.PWTFGQGTK.V 103 (Try/45/-0.0619)<br>95 V.PWTFGQGTKV.E 104 (Pep/30/-0.0407)<br>95 V.PWTFGQGTKVE.I 105 (Pep/52/0.1773)<br>95 V.PWTFGQGTKVEIKRTVA.A 111 (Pep/30/0.013)<br>95 V.PWTFGQGTKVEIKRTVAA.P 112 (Pep/72/0.1271)<br>97 W.TFGQGTKVEIKRT.V 109 (Chy/26/0.0868)<br>97 W.TFGQGTKVEIKRTVAAPSVF.I 116 (Chy/55/-0.1938)<br>99 F.GQGTKVEIKRT.V 109 (Chy/47/0.1589)<br>99 F.GQGTKVEIKRTVAAPSVF.I 116 (Chy/67/-0.0468)<br>99 F.GQGTKVEIKRTVAAPSVF.I 116 (Pep/51/0.1482)<br>102 G.TKVEIKRTVAAPSV.F 115 (Pep/60/0.1782)<br>102 G.TKVEIKRTVAAPSVF.I 116 (Pep/57/0.18)<br>108 K.RTVAAPSVFIFPPSDEQLK.S 126 (Try/74/-0.1079)<br>109 R.TVAAPSVFIFPPSDEQLK.S 126 (Try/97/-0.1022)<br>111 V.AAPSVFIFPPSDEQLK.S 126 (Try/74/-0.1095)<br>112 A.APSVFIFPPSDEQLK.S 126 (Try/64/-0.0996)<br>113 A.PSVFIFPPSDEQLK.S 126 (Try/98/0.0081)<br>117 F.IFPPSDEQLKSGTASVV.C 133 (Chy/29/0.077)<br>117 F.IFPPSDEQLKSGTASVVCL.L 135 (Chy/60/-0.0178)<br>117 F.IFPPSDEQ*LKSGTASVVCL.L Deamidation(Q) 135 (Chy/48/-0.1208)<br>117 F.IFPPSDEQLKSGTASVVCLL.N 136 (Chy/90/0.0204)<br>119 F.PPSDEQLK.S 126 (Try/45/-0.0156)<br>119 F.PPSDEQLKSGTASVVCL.L 135 (Chy/75/0.0677)<br>119 F.PPSDEQL.K 125 (Pep/32/0.1786)<br>127 K.SGTASVVCLLNNFYPR.E 142 (Try/64/0.1039)<br>128 S.GTASVVCLLNNFYPR.E 142 (Try/37/-0.0765)<br>129 G.TASVVCLLNNFYPR.E 142 (Try/58/0.0566)<br>130 T.ASVVCLLNNFYPR.E 142 (Try/47/-0.0063)<br>130 T.ASVVCLLNNFYPR.E 142 (Try/33/0.0035)<br>131 A.SVVCLLNNFYPR.E 142 (Try/53/-0.1648)<br>134 V.CLLNNFYPREAKVQW.K 148 (Chy/52/-0.1399)<br>135 C.LLNNFYPR.E 142 (Try/42/0.1633)<br>135 C.LLNNFYPREAKVQW.K 148 (Chy/46/0.0419)<br>136 L.LNNFYPREAKVQW.K 148 (Chy/62/0.0754)<br>137 L.NNFYPREAKVQW.K 148 (Chy/60/0.0667)<br>140 F.YPREAKVQWKVDNAL.Q 154 (Pep/30/0.1019)<br>140 F.YPREAKVQWKVDNALQSGNSQE.S 161 (Pep/59/-0.1789)<br>144 E.AKVQWKVDNALQSGNSQE.S 161 (Pep/114/0.0407)<br>144 E.AKVQWKVDNALQSGNSQ*E.S Deamidation(Q) 161 (Pep/45/-0.0619)

		144 E.AKVQWKVDNALQSGNSQESV.T 163 (Pep/36/-0.0621)<br>149 W.KVDNALQSGNSQESVTEQD.S 167 (Chy/79/-0.0838)<br>149 W.KVDNALQSGNSQESVTEQDSKDSTY.S 173 (Chy/47/0.1194)<br>149 W.KVDNALQSGNSQE.S 161 (Pep/113/0.1557)<br>150 K.VDNALQSGNSQESVTEQD.S 167 (Try/65/-0.1356)<br>150 K.VDNALQSGNSQESVTEQDSK.D 169 (Try/137/-0.1814)<br>155 L.QSGNSQESVTEQDSK.D 169 (Try/42/-0.0135)<br>156 Q.SGNSQESVTEQDSKDSTY.S 173 (Chy/53/-0.1147)<br>162 E.SVTEQDSKDSTYSL.S 175 (Pep/60/0.1796)<br>162 E.SVTEQDSKDSTYSLSST.L 178 (Pep/71/0.1154)<br>162 E.SVTEQDSKDSTYSLSSTL.T 179 (Pep/60/0.0894)<br>170 K.DSTYSLSSTLTLSK.A 183 (Try/86/-0.0919)<br>173 T.YSLSSTLTLSK.A 183 (Try/48/-0.1599)<br>174 Y.SLSSTLTLSKADYEKHKVY.A 192 (Chy/53/-0.0671)<br>179 T.LTLSKADYEKHKVY.A 192 (Pep/41/0.0046)<br>179 T.LTLSKADYEKHKVYACE.V 195 (Pep/77/0.0028)<br>180 L.TLSKADYEKHKVY.A 192 (Chy/64/0.0599)<br>180 L.TLSKADYEKHKVY.A 192 (Pep/52/0.0208)<br>181 T.LSKADYEKHKVYACE.V 195 (Pep/80/0.0156)<br>182 L.SKADYEKHKVY.A 192 (Chy/37/0.1494)<br>182 L.SKADYEKHKVYACE.V 195 (Pep/87/-0.0276)<br>183 S.KADYEKHKVY.A 192 (Chy/26/0.1834)<br>191 K.VYACEVTHQGLSSPVTK.S 207 (Try/96/0.0346)<br>192 V.YACEVTHQGLSSPVTK.S 207 (Try/113/0.018)<br>193 Y.ACEVTHQGLSSPVTK.S 207 (Try/114/0.0883)<br>193 Y.ACEVTHQGL.S 201 (Chy/35/-0.0328)<br>193 Y.ACEVTHQGLSSPVTK.S 207 (Chy/80/0.1123)<br>193 Y.ACEVTHQGLSSPVTKSF.N 209 (Chy/73/-0.0775)<br>193 Y.ACEVTHQGLSSPVTKSFNRGEC.- 214 (Pep/47/0.1958)<br>194 A.CEVTHQGLSSPVTK.S 207 (Try/75/0.1506)<br>194 A.CEVTHQGL.S 201 (Chy/35/0.0495)<br>195 C.EVTHQGLSSPVTK.S 207 (Try/65/0.0285)<br>196 E.VTHQ*GLSSPVTKS.F Deamidation(Q) 208 (Pep/33/0.1336)<br>196 E.VTHQGLSSPVTKSF.N 209 (Pep/37/0.0566)<br>196 E.VTHQGLSSPVTKSFNRGEC.- 214 (Pep/70/-0.0105)<br>197 V.THQGLSSPVTKSFNRGEC.- 214 (Pep/47/0.1699)<br>202 L.SSPVTKSFNRGEC.- 214 (Chy/44/0.0046)<br>204 S.PVTKSFNRGEC.- 214 (Chy/36/0.0007)
7	78%	1 -.DIQMTQSPSSLSASVGDR.V 18 (Try/133/0.0319)<br>1 -.DIQ*M*TQSPSSLSASVGDR.V Deamidation(Q) Oxidation(M) 18 (Try/60/-0.129)<br>2 D.IQMTQSPSSLSASVGDR.V 18 (Try/112/0.0481)<br>3 I.QMTQSPSSLSASVGDR.V 18 (Try/98/-0.0017)<br>4 Q.MTQSPSSLSASVGDR.V 18 (Try/32/0.1727)<br>8 S.PSSLSASVGDR.V 18 (Try/68/0.1433)<br>19 R.VTITCSASQDISNYLNWYQQKPGK.A 42 (Try/70/-0.1567)<br>21 T.ITCSASQDISN*YLNWYQQKPGK.A 42 (Try/87/-0.1064)<br>24 C.SASQDISNYLNWYQQKPGK.A 42 (Try/62/-0.1233)<br>30 I.SNYLNWYQQKPGK.A 42 (Try/63/-0.0291)

		46 K.VLIYFTSSLHSGVPSR.F 61 (Try/106/-0.0029)<br>47 V.LIYFTSSLHSGVPSR.F 61 (Try/107/0.0593)<br>48 L.IYFTSSLHSGVPSR.F 61 (Try/105/0.0844)<br>49 I.YFTSSLHSGVPSR.F 61 (Try/94/0.1292)<br>50 Y.FTSSLHSGVPSR.F 61 (Try/85/0.1299)<br>51 F.TSSLHSGVPSR.F 61 (Try/70/0.0748)<br>52 T.SSLHSGVPSR.F 61 (Try/34/0.087)<br>88 Y.CQQYSTVPWTFGQGTK.V 103 (Try/59/-0.1649)<br>95 V.PWTFGQGTK.V 103 (Try/31/0.0133)<br>108 K.RTVAAPSVFIFPPSDEQLK.S 126 (Try/41/-0.1713)<br>109 R.TVAAPSVFIFPPSDEQLK.S 126 (Try/78/0.1478)<br>111 V.AAPSVFIFPPSDEQLK.S 126 (Try/70/0.0311)<br>112 A.APSVFIFPPSDEQLK.S 126 (Try/52/-0.0592)<br>113 A.PSVFIFPPSDEQLK.S 126 (Try/64/0.0335)<br>119 F.PPSDEQLK.S 126 (Try/59/0.0082)<br>127 K.SGTASVVCLLNNFYPR.E 142 (Try/71/0.0851)<br>127 K.SGTASVVCLLN*NFYPR.E 142 (Try/55/0.0579)<br>131 A.SVVCLLNNFYPR.E 142 (Try/43/-0.1028)<br>150 K.VDNALQSGNSQESVTEQDSK.D 169 (Try/140/-0.0138)<br>152 D.NALQSGNSQESVTEQDSK.D 169 (Try/63/-0.0478)<br>155 L.QSGNSQESVTEQDSK.D 169 (Try/53/-0.1219)<br>159 N.SQESVTEQDSK.D 169 (Try/48/-0.0609)<br>170 K.DSTYSLSSTLTLSK.A 183 (Try/91/0.0805)<br>191 K.VYACEVTHQGLSSPVTK.S 207 (Try/98/0.0832)<br>192 V.YACEVTHQGLSSPVTK.S 207 (Try/98/0.0506)<br>193 Y.ACEVTHQGLSSPVTK.S 207 (Try/100/0.0611)<br>194 A.CEVTHQGLSSPVTK.S 207 (Try/81/0.0826)<br>195 C.EVTHQGLSSPVTK.S 207 (Try/62/0.1543)

Table S1B

MS/MS results of 4 spots from Avastin (MASCOT) identified by Orbitrap

Avastin was separated by 2-DE and identified by LTQ-Orbitrap Velos ETD MS/MS, using the MASCOT search engine.

spot	coverage	Identified Peptide (enzyme/ion score/mass error[ppm])
2	76%	1 -.EVQLVESGGGLVQPGGSLR.L 19(Trypsin/129/0)<br>20 R.LSCAASGYTFTNYGM*NWVR.Q Oxidation(M) 38(Trypsin/112/0)<br>44 K.GLEWVGWINTYTGEPTYAADFK.R 65(Trypsin/47/0)<br>68 R.FTFSLDTSK.S 76(Trypsin/51/0)<br>68 R.FTFSLDTSKSTAYLQM*NSLR.A Oxidation(M) 87(Trypsin/37/-1)<br>68 R.FTFSLDTSKSTAYLQM*NSLR.A Oxidation(M) 87(Trypsin/56/0)<br>77 K.STAYLQM*NSLR.A Oxidation(M) 87(Trypsin/64/0)<br>88 R.AEDTAVYYCAK.Y 98(Trypsin/69/0)<br>128 K.GPSVFPLAPSSK.S 139(Trypsin/57/0)<br>128 K.GPSVFPLAPSSKSTSGGTAALGCLVK.D 153(Trypsin/44/1)<br>140 K.STSGGTAALGCLVK.D 153(Trypsin/98/-1)<br>217 K.VDKKVEPK.S 224(Trypsin/26/0)<br>220 K.KVEPKSCDK.T 228(Trypsin/23/0)<br>225 K.SCDKTHTCPPCPAPELLGGPSVFLFPPKPK.D 254(Trypsin/64/-1)<br>229 K.THTCPPCPAPELLGGPSVFLFPPKPK.D 254(Trypsin/59/-1)<br>255 K.DTLMISR.T 261(Trypsin/34/0)<br>255 K.DTLM*ISR.T Oxidation(M) 261(Trypsin/45/0)<br>255 K.DTLM*ISRTPEVTCVVVDVSHEDPEVK.F Oxidation(M) 280(Trypsin/40/-1)<br>262 R.TPEVTCVVVDVSHEDPEVK.F 280(Trypsin/90/0)<br>281 K.FNWYVDGVEVHNAK.T 294(Trypsin/73/0)<br>295 K.TKPREEQYNSTYR.V 307(Trypsin/40/1)<br>299 R.EEQYNSTYR.V 307(Trypsin/37/0)<br>308 R.VVSVLTVLHQDWLNGK.E 323(Trypsin/44/1)<br>308 R.VVSVLTVLHQDWLNGK.E 323(Trypsin/37/1)<br>329 K.VSNKALPAPIEK.T 340(Trypsin/49/0)<br>333 K.ALPAPIEK.T 340(Trypsin/26/1)<br>341 K.TISKAKGQPR.E 350(Trypsin/44/0)<br>351 R.EPQVYTLPPSREEMTK.N 366(Trypsin/49/-1)<br>351 R.EPQVYTLPPSREEM*TK.N Oxidation(M) 366(Trypsin/48/-1)<br>362 R.EEMTKNQVSLTCLVK.G 376(Trypsin/61/0)<br>362 R.EEM*TKNQVSLTCLVK.G Oxidation(M) 376(Trypsin/46/0)<br>367 K.NQVSLTCLVK.G 376(Trypsin/51/0)<br>377 K.GFYPSDIAVEWESNGQPENNYK.T 398(Trypsin/68/0)<br>399 K.TTPPVLDSDGSFFLYSK.L 415(Trypsin/74/1)<br>416 K.LTVDKSR.W 422(Trypsin/35/0)<br>423 R.WQQGNVFSCSVMHEALHNHYTQK.S 445(Trypsin/50/-1)
4	83%	1 -.EVQLVESGGGLVQPGGSLR.L 19(Trypsin/102/-1)

		20 R.LSCAASGYTFTNYGM*NWVR.Q Oxidation(M) 38(Trypsin/35/0)<br>67 R.RFTFSLDTSK.S 76(Trypsin/44/0)<br>68 R.FTFSLDTSK.S 76(Trypsin/50/0)<br>77 K.STAYLQMNSLR.A 87(Trypsin/51/1)<br>77 K.STAYLQM*NSLR.A Oxidation(M) 87(Trypsin/59/1)<br>88 R.AEDTAVYYCAK.Y 98(Trypsin/81/0)<br>128 K.GPSVFPLAPSSK.S 139(Trypsin/53/0)<br>140 K.STSGGTAALGCLVK.D 153(Trypsin/80/0)<br>154 K.DYFPEPVTVSWNSGALTSGVHTFPAVLQSSGLYSLSSVVTVPSSSLGTQTYICNVNHKPSNTK.V 216(Trypsin/30/-1)<br>225 K.SCDKTHTCPPCPAPELLGGPSVFLFPPKPK.D 254(Trypsin/68/-1)<br>229 K.THTCPPCPAPELLGGPSVFLFPPKPK.D 254(Trypsin/59/0)<br>255 K.DTLM*ISR.T Oxidation(M) 261(Trypsin/44/0)<br>262 R.TPEVTCVVVDVSHEDPEVK.F 280(Trypsin/48/1)<br>281 K.FNWYVDGVEVHNAK.T 294(Trypsin/55/-1)<br>299 R.EEQYNSTYR.V 307(Trypsin/32/0)<br>308 R.VVSVLTVLHQDWLNGK.E 323(Trypsin/60/0)<br>329 K.VSNKALPAPIEK.T 340(Trypsin/44/0)<br>341 K.TISKAKGQPR.E 350(Trypsin/38/0)<br>351 R.EPQVYTLPPSR.E 361(Trypsin/41/0)<br>351 R.EPQVYTLPPSREEM*TK.N Oxidation(M) 366(Trypsin/47/-1)<br>367 K.NQVSLTCLVK.G 376(Trypsin/53/0)<br>399 K.TTPPVLDSDGSFFLYSK.L 415(Trypsin/40/-2)<br>399 K.TTPPVLDSDGSFFLYSK.L 415(Trypsin/48/0)<br>416 K.LTVDKSR.W 422(Trypsin/34/1)<br>423 R.WQQGNVFSCSVMHEALHNHYTQK.S 445(Trypsin/32/0)<br>423 R.WQQGNVFSCSVM*HEALHNHYTQK.S Oxidation(M) 445(Trypsin/34/2)
6	100%	1 -.DIQMTQSPSSLSASVGDR.V 18(Trypsin/Ions/0)<br>19 R.VTITCSASQDISNYLNWYQQKPGK.A 42(Trypsin/Ions/0)<br>19 R.VTITCSASQDISNYLNWYQQKPGKAPK.V 45(Trypsin/Ions/-1)<br>43 K.APKVLIYFTSSLHSGVPSR.F 61(Trypsin/Ions/0)<br>46 K.VLIYFTSSLHSGVPSR.F 61(Trypsin/Ions/0)<br>62 R.FSGSGSGTDFTLTISSLQPEDFATYYCQQYSTVPWTFGQGTK.V 103(Trypsin/Ions/1)<br>104 K.VEIKRTVAAPSVFIFPPSDEQLK.S 126(Trypsin/Ions/0)<br>108 K.RTVAAPSVFIFPPSDEQLK.S 126(Trypsin/Ions/0)<br>127 K.SGTASVVCLLNNFYPR.E 142(Trypsin/Ions/0)<br>143 R.EAKVQWK.V 149(Trypsin/Ions/0)<br>150 K.VDNALQSGNSQESVTEQDSK.D 169(Trypsin/Ions/0)<br>150 K.VDNALQSGNSQESVTEQDSKDSTYSLSSTLTLSK.A 183(Trypsin/Ions/-1)<br>170 K.DSTYSLSSTLTLSK.A 183(Trypsin/Ions/-1)<br>170 K.DSTYSLSSTLTLSKADYEK.H 188(Trypsin/Ions/-1)<br>184 K.ADYEKHKVYACEVTHQGLSSPVTK.S 207(Trypsin/Ions/1)<br>189 K.HKVYACEVTHQGLSSPVTK.S 207(Trypsin/Ions/-1)<br>189 K.HKVYACEVTHQGLSSPVTKSFNR.G 211(Trypsin/Ions/0)<br>191 K.VYACEVTHQGLSSPVTK.S 207(Trypsin/Ions/1)<br>191 K.VYACEVTHQGLSSPVTK.S 207(Trypsin/Ions/2)<br>191 K.VYACEVTHQGLSSPVTKSFNR.G 211(Trypsin/Ions/0)<br>208 K.SFNRGEC.- 214(Trypsin/Ions/0)

7	95%	1 -.DIQMTQSPSSLSASVGDR.V 18(Trypsin/114/0)<br>1 -.DIQM*TQSPSSLSASVGDR.VoxidationM) 18(Trypsin/101/0)<br>19 R.VTITCSASQDISNYLNWYQQKPGK.A 42(Trypsin/97/-1)<br>19 R.VTITCSASQDISNYLNWYQQKPGKAPK.V 45(Trypsin/34/-1)<br>43 K.APKVLIYFTSSLHSGVPSR.F 61(Trypsin/43/-1)<br>46 K.VLIYFTSSLHSGVPSR.F 61(Trypsin/100/0)<br>62 R.FSGSGSGTDFTLTISSLQPEDFATYYCQQYSTVPWTFGQGTK.V 103(Trypsin/47/1)<br>108 K.RTVAAPSVFIFPPSDEQLK.S 126(Trypsin/68/0)<br>127 K.SGTASVVCLLNNFYPR.E 142(Trypsin/85/-1)<br>150 K.VDNALQSGNSQESVTEQDSK.D 169(Trypsin/112/-1)<br>150 K.VDNALQSGNSQESVTEQDSK.D 169(Trypsin/126/0)<br>150 K.VDNALQSGNSQESVTEQDSKDSTYSLSSTLTLSK.A 183(Trypsin/95/0)<br>170 K.DSTYSLSSTLTLSK.A 183(Trypsin/71/0)<br>170 K.DSTYSLSSTLTLSKADYEK.H 188(Trypsin/94/1)<br>189 K.HKVYACEVTHQGLSSPVTK.S 207(Trypsin/83/0)<br>191 K.VYACEVTHQGLSSPVTK.S 207(Trypsin/84/0)<br>191 K.VYACEVTHQGLSSPVTKSFNR.G 211(Trypsin/86/0)<br>208 K.SFNRGEC.- 214(Trypsin/44/0)
