# Supplementary material for: Proteins from Avastin® (bevacizumab) Show Tyrosine Nitrations for which the Consequences Are Completely Unclear
Source: PLoS One. 2012 Apr 16;7(4):e34511. doi: 10.1371/journal.pone.0034511 (PMC3327692; doi:10.1371/journal.pone.0034511)
Supplement: Table S2 — The modifications revealed by the Modiro search engine from HCT and Orbitrap data. (PDF) [file pone.0034511.s004.pdf]

Table S2A

The modifications revealed by the Modiro search engine from HCT data

Spot	CID	ETD
1	Substitution: L4H, P277S<br>2-amino-3-oxo-butanoic_acid: S140<br>Acetylation: S430<br>Carbamylation: E1<br>Deamidation: N321<br>Dihydroxy: R38,T372, T373<br>Dimethylation: P33,P34<br>Methylation: S7,H291<br>Oxidation: M34,M83	Substitution: E1K, M34V<br>Carbamylation: E1<br>Methylation: H6
2	Substitution: N52D, P238T, V48D, C266G, E264K, D271H, I51N, L320P, N292D, N303D, N440D<br>Acetylation: S260<br>Amino: Y54, Y155<br>Biotinylation: K252<br>Deamidation: R19, R38, R87, Q115, R298, R307, Q317, N321, N390, N395<br>Dihydroxy: P233, C235, T266, Y284<br>Dimethylation: P129, P233<br>Ethylation: K415<br>Hydroxylation: P14, N52, N292<br>Methylation: S17, S21, T53, K153, E158, S260, T266,E278, H291, D407, C431, S432,<br>Oxidation: M4, M34, M83<br>Sodiated: E6	Substitution: D271H, N292D, Q317E, D405H, D407H, N440D<br>Deamidation: Q115, Q317, N321<br>Dimethylation: P233<br>L-allysine: K280<br>Methylation: S21, H274, E278, D407, S409<br>Oxidation: M34, M83<br>Sodiated: E6
3	Substitution: V48D, N52D, K139E, P236T, N440D<br>Acetylation: S432<br>Amino: Y284<br>Deamidation: R19, Q115, R307, N321, N390<br>Dihydroxy: Y284<br>Dimethylation: P233, P234<br>Ethylation: K415<br>Hydroxylation: D318<br>L-allysine: K252<br>Methylation: S21, E158, E264, E275, E278, H316, C431, S432, H435	Substitution: D407H<br>Amino: Y284<br>Deamidation: Q115, N321<br>Dihydroxy: Y284<br>Hexosamine: T91<br>Hydroxylation: D73, P263<br>Methylation: S21, E275, K280, H316<br>Oxidation: M34

	Oxidation: M83, M335, M434<br>Trimethyl-OH: K139	
4	Substitution: F429I<br>Deamidation : N321<br>Dihydroxy: Y284<br>Hexose : C431<br>L-allysine: K139<br>Methylation: E6<br>Oxidation: M34, M83, M434	Dihydroxy: Y284<br>Hydroxylation: P263<br>Oxidation :M34
5	Substitution: D120H,D151H， D1H， D1N， D28H， I117N， M4R， T5R， N137D， N31D， N34D， Q155E， S30A， V196D， K126E<br>Acetylation: D1<br>Carbamylation: D1, K42, K45, K149<br>Deamidation: R18, R142, Q199<br>Dihydroxy: T5, T20, T22, Y32, T197<br>Dimethylation: P204<br>GlyGLy: K190<br>Hydroxylation: D1<br>Hexosamine: T51, N210<br>Hexose: K183<br>Methylation: H55, S121, D122, Q124, S127, Q160, E161, E165, E195<br>N-pyruvic acid 2-iminyl: K190<br>Phosphorylation: S127<br>Pyruvat: C23	Substitution: D1N<br>Carbamylation: D1, K45<br>Deamidation: R108, R142, Q199<br>Dihydroxy: T197<br>Methylation: H55, E165<br>Oxidation: M4
6	Substitution: D122H, D151H, D170N, D1H, D1N, I117N, I29N, I48M, L154F, D167H, K126E<br>Acetylation: K190<br>Amino: Y192<br>Carboxyethyl: K149<br>Dihydroxylation: T197<br>Deamidation: R18, Q27, N34,R 108, N137, R142, Q199<br>Hexosamine: T51<br>Hydroxylation: D1<br>L-allysine: K39<br>Methylation: S9, S10, Q27, N31, N34, S52, S53, H55, S121, D122, E123, S127, S131, S156, N158, E165, E195<br>Nitro: Y186, Y192<br>Oxidation: M4	Substitution: A193E, D1N<br>Deamidation: R108, R142, Q199<br>Methylation: S52, S53, S56, T164<br>Oxidation: M4

	Trimethyl-OH: K190	
7	Substitution: D1H, D1N, D28H, D122H, I48N, D151H, D170H, D170N<br>Amino: Y192<br>Deamidation: Q3, R18, Q27, N31, N34, N137, Q199<br>Dihydroxylation: Y36, P44, K45, Y49, K190, T197<br>Diphthamide: H189<br>Hydroxylation: K39<br>Methylation: Q3, Q6, S7, Q27, T51, E123, S127, E165, E195, Q199<br>Oxidation: M4	Substitution: V191G<br>Deamidation: R285<br>Methylation: Q124, E165, Q199

Table S2B

The modifications revealed by the Modiro search engine from Orbitrap data

Spot	Modification
2	Substitution: N440D, L320Q, N367D, G287D, N395D, N292D, G322D, F29I, G144D, C230G, S85N, A333E, G149D, A79S, A97E, N331D, G322D, Y94D, F70Y, L320Q, N303D, N292D, Q317E, K346Q, F70Y, M258T, G149D, A97E, N331D, F70Y, T372N, S85N, F70Y, N303D<br>Acetylation: S140<br>Carbamylation: K139, K224, K366, K420<br>Deamidation: R87, N321, N390<br>Dihydroxy: S7, Y32, R38, Y80, Y94, K153, P188, Y284, K323, S389, S430<br>GlyGLy: K332, , K420<br>Hexose: K139, R289<br>Hydroxylation: N282, P234, N390<br>Kynurenin: W36, W283, W319<br>L-allysine: K366<br>Methylation: E6, E89, K98, T266, E264, E275, E289, E299, T372<br>Nitro: Y27, Y80, Y355, Y413<br>Oxidation: M22, M34, M83, M258, M364, M434<br>Sulphone: M34
4	Substitution: N440D, L320Q, N367D, G287D, N395D, N292D, G322D, F29I, G144D, C230G, S85N, A333E, G149D, A79S, A97E, N331D, G322D, Y94D, F70Y, L320Q, N303D, N292D, Q317E, K346Q, F70Y, M258T, G149D, A97E, N331D, F70Y, T372N, S85N, F70Y, N303D<br>Acetylation: K328<br>Carbamylation: K332, K340<br>Deamidation: R87, N321<br>Dihydroxy: R87, Y284<br>GlyGLy: K98, K42<br>Hexose: K98<br>Hydroxylation: D73, N282<br>Kynurenin: W283<br>Methylation: E6, E89, E289, T372<br>Oxidation: M34, M83, M258, M364<br>Sulphone: M83
6	Substitution: K207R, T197S, F50Y, N158D, L154S, L154Q, N137D, T197S, R19K, N34D, Q199R, T197S, F50Y, N158K, N137D, R18K<br>Amino: Y192

	Acetylation: S7, K149, K207<br>Carbamylation: K207<br>Deamidation: R142, Q199<br>Dihydroxy: Y36,S56,K149,S156,S208,<br>GlyGLy: K190, K207<br>Hexose: W148, K183<br>Hydroxylation: D122, N137, K183<br>L-allysine: K207<br>Methylation: S121, D122, E123, E165, E195<br>Nitro: Y36, Y49, Y140,Y173, Y192<br>Oxidation M4<br>Sulphone: M4
7	Substitution: K207R, T197S, F50Y, N158D, L154S, L154Q, N137D, T197S, R19K, N34D, Q199R, T197S, F50Y, N158K, N137D, R18K<br>Acetylation: K149,K190,K207<br>Carbamylation: K190, K207<br>Deamidation: R142, Q199<br>Dihydroxy: Y36, K149, T197<br>GlyGLy: K190<br>Hexose: K183<br>Hydroxylation: K39, N137, D170, K190<br>L-allysine: K207<br>Methylation: E123, E165, E195<br>Nitro: Y49, Y173, Y192<br>Oxidation: M4<br>Sulphone: M4
